# Paleogenomics reveals leprosy at the late antique burial site of Fallward in northern Germany

**DOI:** 10.1016/j.isci.2026.117877

**Published:** 2026-10-15

**Authors:** Daniel Anton Myburgh, Nicolas Antonio da Silva, Joanna H. Romeyer-Dherbey, Silke Grefen-Peters, Hauke Jöns, Christina Peek, Almut Nebel, Ben Krause-Kyora

**Affiliations:** 1 Institute of Clinical Molecular Biology, Kiel University, 24105 Kiel, Germany; 2 Department of Genetics, School of Medicine, Yale University, New Haven, CT 06520, USA; 3 Ossatura, 38104 Braunschweig, Germany; 4 Niedersächsisches Institut für historische Küstenforschung, 26382 Wilhelmshaven, Germany

**Keywords:** ancient DNA, Roman Iron Age, Migration Period, *Mycobacterium leprae*, mobility, cultural

## Abstract

The coastal settlement of Fallward in northwestern Germany (3^rd^–5^th^ century CE) is renowned for exceptionally preserved wooden and textile grave goods, yet the genetic and pathogen landscape of its inhabitants has remained unstudied. We conducted metagenomic analyses on skeletal samples from 11 individuals to investigate their genetic affinities and infectious disease history. Three individuals yielded sufficient data for population genetic analysis: two clustered with northwestern European Iron Age and Anglo-Saxon groups, while one adult male might have originated from a Roman frontier population. This man was also infected with a strain of *Mycobacterium leprae* belonging to phylogenetic branch 4 and was buried in a boat accompanied by Roman-influenced grave goods, including carved furniture and a military belt. Together, these findings point to possible long-distance connections linking remote communities, such as Fallward, to the wider Roman world during Late Antiquity.

## Highlights

• Human genomes analyzed from the Iron Age and Migration Period in northern Germany
• One *M. leprae*-infected man was buried with Roman-associated goods
• He showed genetic affinity to a Roman Danubian frontier population
• Findings highlight a broad Roman sphere of influence and long-distance mobility

## Graphical abstract

**Figure:**
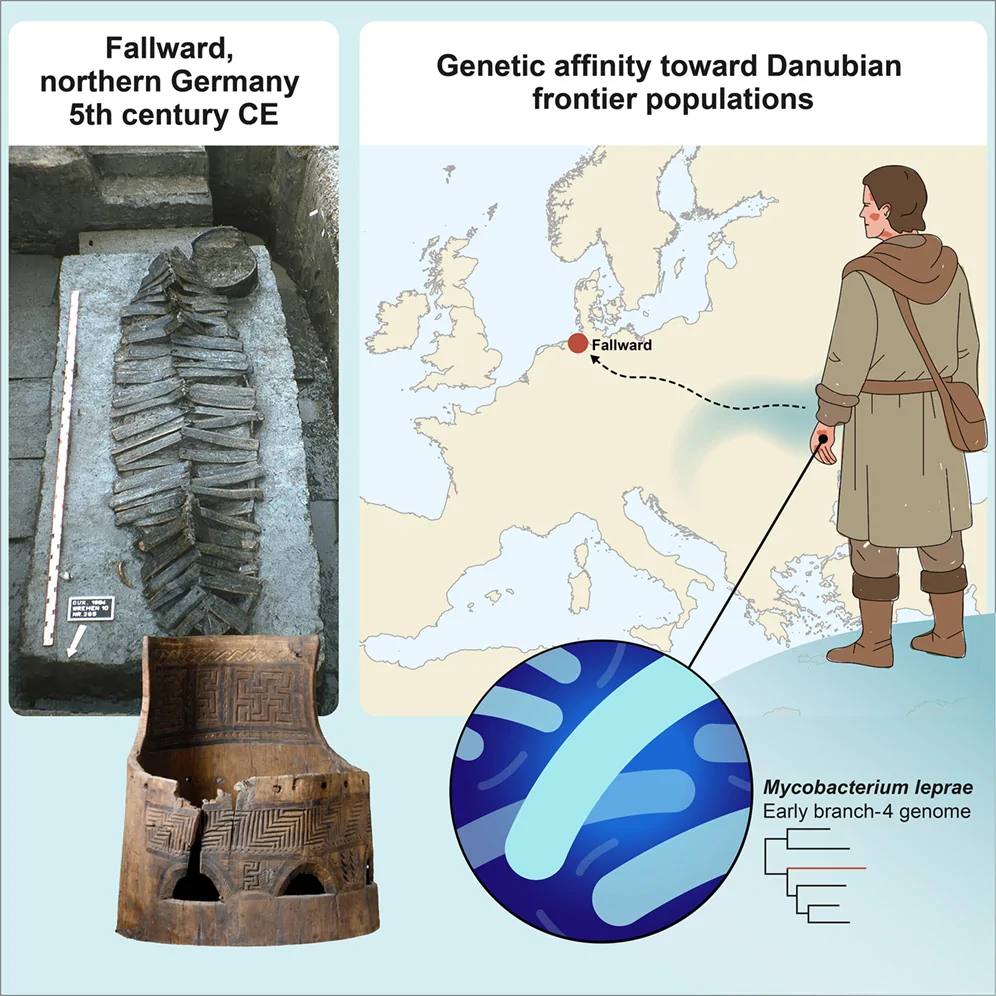

## Teaser

Microbiology; Paleobiology; Paleogenetics; Anthropology

## Introduction

The archeological settlement of Fallward is situated in the Elbe-Weser River triangle in present-day northwestern Germany. It was established atop an artificial dwelling mound, known as a *terp* or *wurt*, constructed on the sandy and gravel-rich deposits of a naturally formed beach ridge that runs parallel to the North Sea coast^1^ (Figure 1). At a time when dikes had not yet been built, these mounds offered safe ground against storm surges, high tides, and flooding—frequent threats in the unpredictable maritime environment.^2^ Despite these external hazards, the fertile soil of the surrounding marshland made agriculture both possible and profitable. Fallward is one of eight settlements in the Elbe-Weser River triangle that was founded during the 1^st^ century BCE^3^ (Figure 1). Preliminary archeological investigations have identified several distinct phases of mound elevation and habitation at the site. However, the settlement gradually declined over the course of the 5^th^ century CE and was likely fully abandoned by the end of it.^4^ This period also saw a marked decrease in settlement activity in many neighboring areas of the North Sea coastal zone, a trend generally linked to migrations to Great Britain noted in contemporaneous written sources.^5^ Consistent with this, recent paleogenomic research has revealed significant introgression from continental northern Europeans into the early Anglo-Saxon gene pool in England.^6^

**Figure 1:**
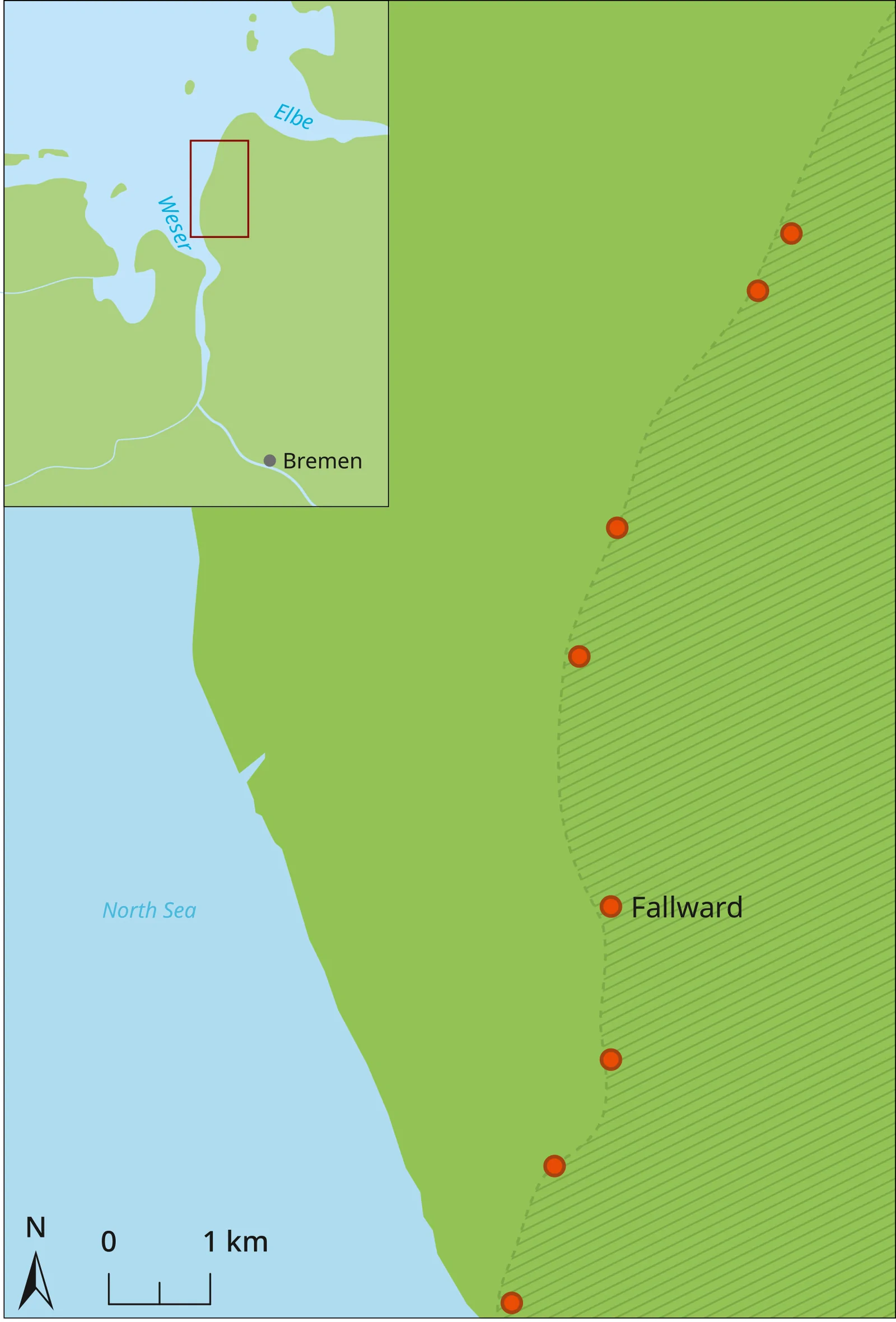
Map showing the location of the dwelling mound Fallward and seven others in northwestern Germany Red circles indicate the locations of the dwelling mound sites. They were built along the ancient coastline (denoted by a line). The hatched area indicates the marshland during the 4^th^ and 5^th^ centuries.

Excavations carried out in the 1990s uncovered a cemetery near the Fallward settlement, which was in use during its final phase—from the end of the 3^rd^ to the late 5^th^ century CE. These dates fall within the Late Roman Iron Age and Migration Period, as defined in northern European archeology. The cemetery comprised around 200 cremation burials and 60 inhumation graves.^4^ Particularly in the northwestern part of the site, many grave pits were dug deep into the fossil marsh clay. In these constantly damp conditions, a wide range of grave goods made from various materials was remarkably well preserved. In addition to different types of coffins, the finds included bedding or upholstery and covers made of plant materials, furs, and textiles; numerous layers of clothing fabrics and related accessories; components of household items made of wood and ceramics; and even some elaborately decorated furniture^4^^,^^7^ (Figures 2A–2C).

**Figure 2:**
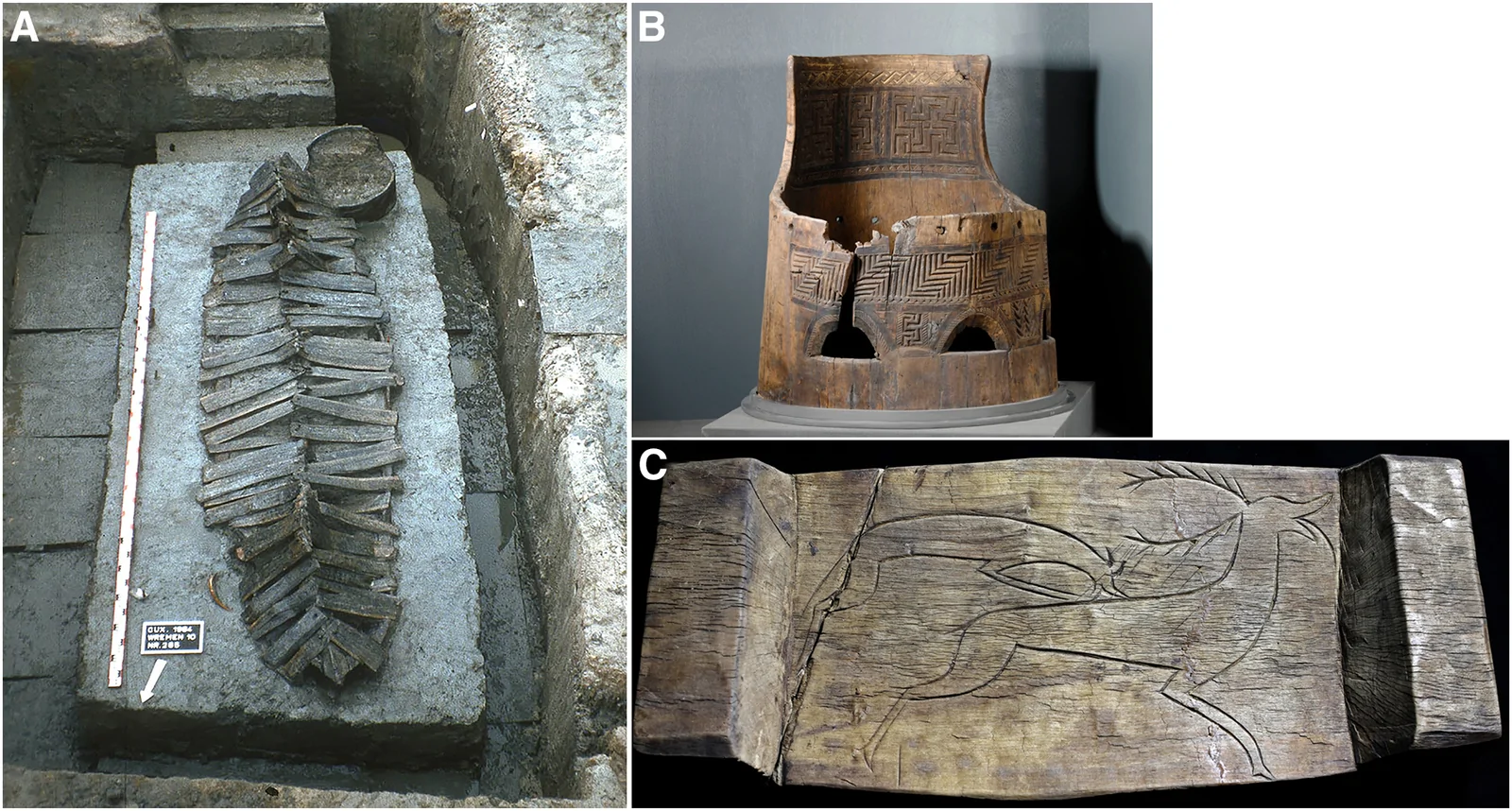
Boat burial and associated furnishings from grave 265 The man in grave 265 was interred in a boat over four meters in length, which was covered with wooden panels forming a roof-like structure (A). The grave also contained a richly decorated chair made of an alder trunk (B) and a footstool with a hunting scene on its underside (C), for which there are numerous comparisons from the Roman Empire (photos: R. Kiepe, Niedersächsisches Institut für historische Küstenforschung).

Thanks to their exceptional preservation, the grave goods from Fallward rank among the most significant burial finds in central Europe from the Late Iron Age and the Migration Period. They have provided valuable insights into the daily life, burial practices and socio-cultural organization of the population living along the North Sea coast during this time. The burial site, together with the grave goods selected to represent the deceased, appears to reflect a society that was very conscious of tradition and remained largely settled in one place. A few particularly rich graves containing foreign items, mainly of Mediterranean origin, were previously associated with a privileged status.^4^^,^^8^

Despite the significance of the site, the human remains from Fallward have not yet been examined using paleogenomic methods. As a result, questions about the genetic makeup of the burial community have remained unanswered. To fill this gap, we performed metagenomic analyses on skeletal samples from 11 individuals. Our investigation focused on exploring their genetic affinities with contemporaneous populations across Europe.

## Results

### Genomic analyses uncover evidence for possible non-local ancestry in one individual

In this study, we isolated DNA from 12 skeletal samples obtained from 11 individuals buried near the dwelling mound settlement Fallward in present-day Germany (Table 1). The DNA extracts were subjected to shotgun sequencing. For eight individuals, the genetic sex could be determined. We identified seven males and one female (Table 1; Figure S1). In three adults for whom osteological and genetic sex assessments were available (G14, G265, and G407), the results were consistent across methods. For three children with genetic and archeological sex determinations, one showed concordant results (G250), while the other two did not (G224 and G282) (Table 1). Kinship relationships within the Fallward group could not be investigated due to low genomic coverage.

**Table 1:** Individuals from Fallward analyzed in this study

Individuals from grave no.	Skeletal element	Age at death (years)^a^	Osteological sex^a^	Archeological sex^a^^,^^b^	Genetic sex
G14	petrous bone	adult	M	M	M
G223	petrous bone	child	–	?	M
G224	petrous bone	child	–	F	M
G250	petrous bone	3	F	F	F
G265	calcaneus, molar	50	M	M	M
G282	petrous bone	1.5	?	F	M
G284	femur	45–55	F	F	–
G407	femur	40	M	M	M
G439	femur	adult	–	?	–
G452	femur	adult	F	F	–
G468	femur	child	–	?	M

a Age at death and osteological/archeological sex as reported previously.^4^^,^^9^

b Archeological sex determination based on type of burial goods. For individuals marked in bold, population genetic analyses could be performed. M = male, F = female, – = analysis not possible, and ? = sex uncertain.

Three samples yielded data of sufficient quality for population genetic analyses (G14, G250, and G265). In a principal component analysis (PCA) plot, G14 and G250 clustered within the variation observed in present-day northwestern Europeans (Figure 3). In contrast, G265 grouped along the genetic cline associated with southeastern European populations (Figure 3), indicating a potentially different ancestral background. Projection of the Fallward individuals onto a PCA including ancient reference populations showed that G14 and G250 most closely aligned with Iron Age and early medieval groups, in particular exhibiting greater affinity to British Iron Age individuals than to those from the German Iron Age (Figure 4). G265 instead clustered with Roman Iron Age and Migration Period individuals from the central and southern Danubian frontier (Croatia, Slovenia, Hungary, and Serbia) (Figure 4). Spatiotemporal interpolation of genetic affinities around 500 CE, corresponding to approximately the archeological date of G265, identified multiple regions of elevated similarity probability, including southern Scandinavia, although the highest values extended across eastern central Europe, with a secondary hotspot in the Pontic steppe (Figure 5). The highest probability location was over 1,400 km southeastwards from Fallward, near the Roman frontier (Figure 5). Consistent with these results, individual G265 carried Y chromosome haplogroup G, a lineage that is most commonly found in the Caucasus today, although it also occurs at lower frequencies throughout Europe^11^ (Table S1). However, complementary analyses (outgroup f_3_ and f_4_ statistics, unsupervised admixture, and qpAdm modeling) did not identify a discrete ancestry profile distinguishing G265 from the other Fallward individuals (Figures S2–S4; Table S2).

**Figure 3:**
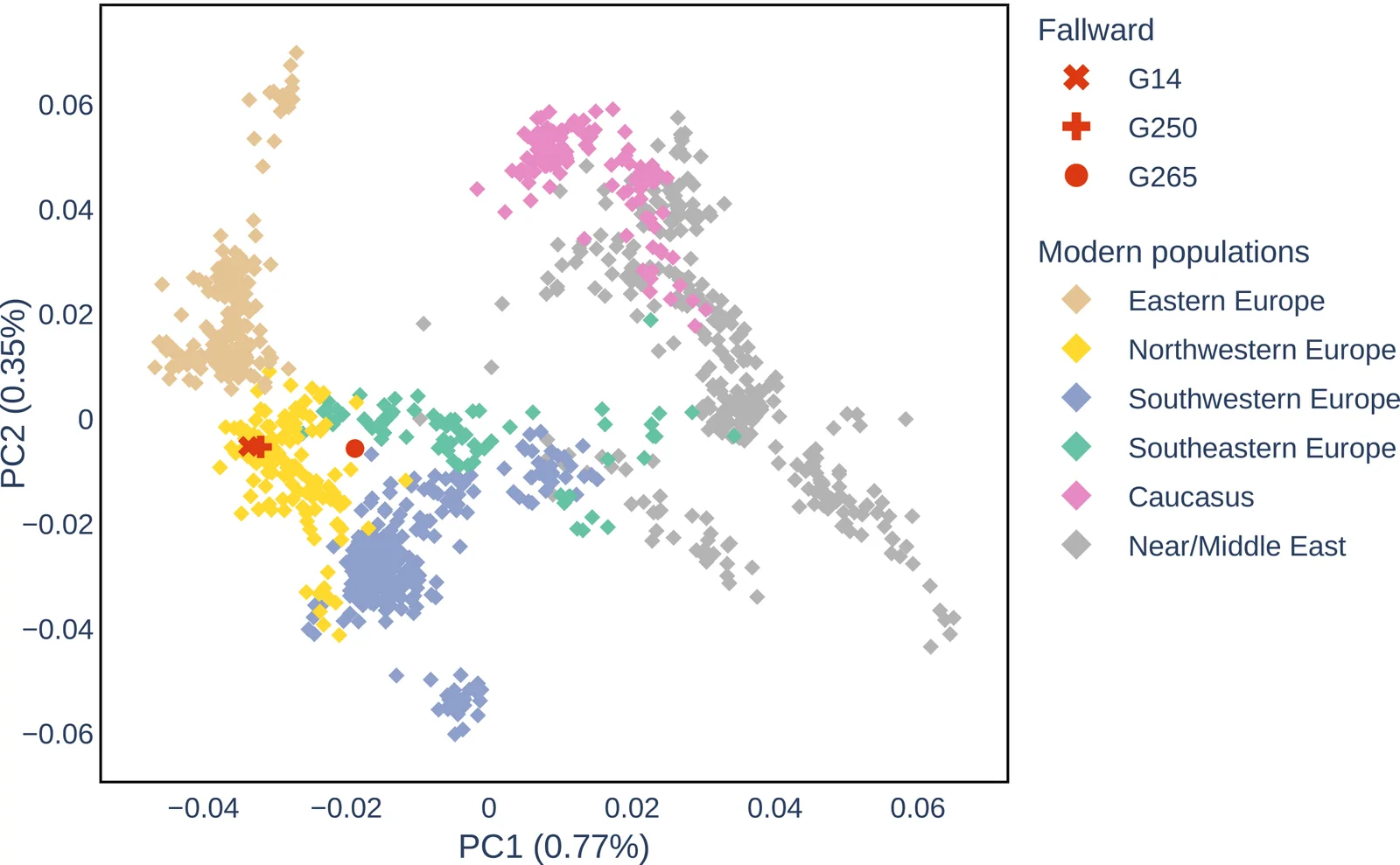
Principal component analysis of the three Fallward individuals projected onto the genetic variation of modern West Eurasians Axis labels indicate the proportion of variance explained by each principal component.

**Figure 4:**
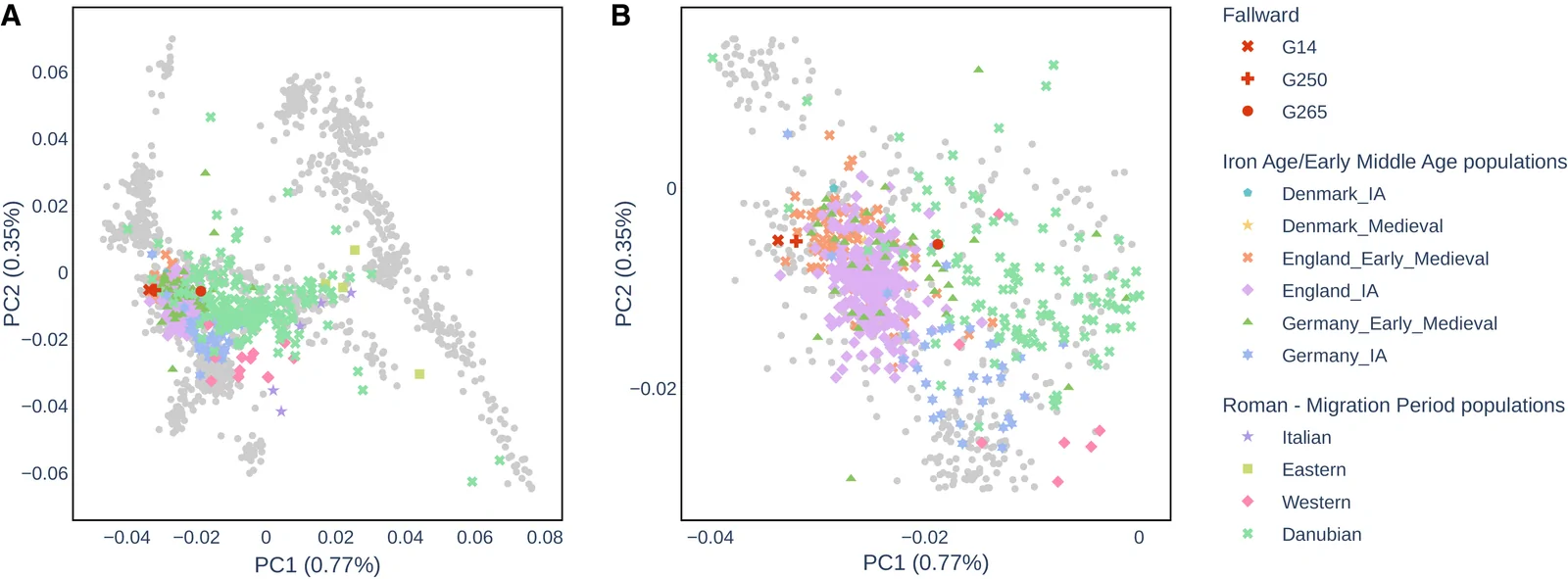
Principal component analysis of the Fallward individuals as well as published Iron Age, Migration Period, and medieval samples (A) The ancient individuals are projected onto the variation of present-day West-Eurasian populations (light gray). (B) Zoomed-in view of the space occupied by the ancient samples. Axis labels indicate the proportion of variance explained by each principal component. Abbreviations: IA, Iron Age.

**Figure 5:**
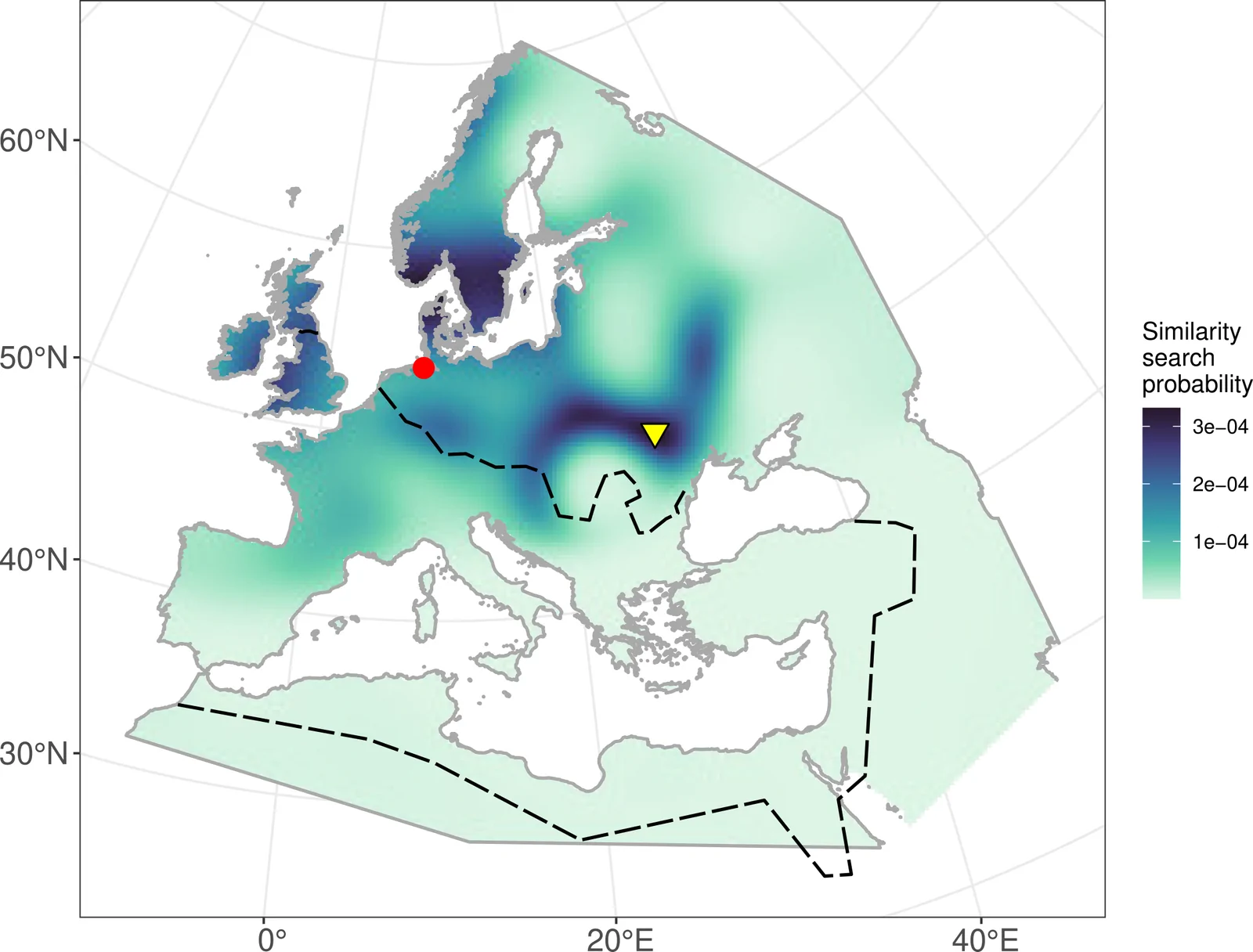
Spatiotemporal interpolation of genetic affinities for G265 around 500 CE using mobest Colors represent the interpolated probability of genetic similarity across the study region based on the first three principal components. The red circle marks the location of Fallward, while the yellow triangle indicates the location of the maximum of the interpolated probability surface. The dashed line represents an approximate reconstruction of the Roman frontier based on the Digital Atlas of the Roman Empire.^10^

### Ancient leprosy genome from Fallward reveals early branch-4 diversity

Metagenomic pathogen screening revealed the presence of *Mycobacterium leprae* (*M. leprae*) DNA in two samples (tooth and calcaneus bone) from G265. To investigate these *M. leprae* reads, mapping was conducted using the *M. leprae* TN (National Center for Biotechnology Information [NCBI] RefSeq: NC_002677.1) reference genome. Mapping statistics indicated high coverage of the reference genome, with a 4× coverage of more than 99% and a mean depth of 58.3 (Table S3). Moreover, deamination patterns observed for the aligned reads were characteristic of ancient DNA (Figure S5).

Thereafter, we constructed a phylogeny consisting of 44 ancient and 185 modern *M. leprae* strains, representing all major branches^12^^,^^13^^,^^14^^,^^15^^,^^16^^,^^17^^,^^18^^,^^19^^,^^20^^,^^21^^,^^22^^,^^23^^,^^24^^,^^25^^,^^26^^,^^27^^,^^28^^,^^29^^,^^30^^,^^31^^,^^32^^,^^33^^,^^34^ (Figure 6). The tree revealed that the G265 *M. leprae* genome was ancestral to the known diversity of branch 4, being predated only by Abusir1630 from an Egyptian mummy^13^ (Figure 6). Subsequent single nucleotide polymorphism (SNP) effect analysis of the G265 *M. leprae* strain identified eight high-impact SNPs (Table S4), seven of which were frameshifts and one causing a premature stop codon. Although these variants are predicted to strongly disrupt protein function (for example through truncation, loss of function or nonsense mediated decay), their widespread presence across ancient and modern strains indicates they are not novel disruptive events unique to the G265 strain.

**Figure 6:**
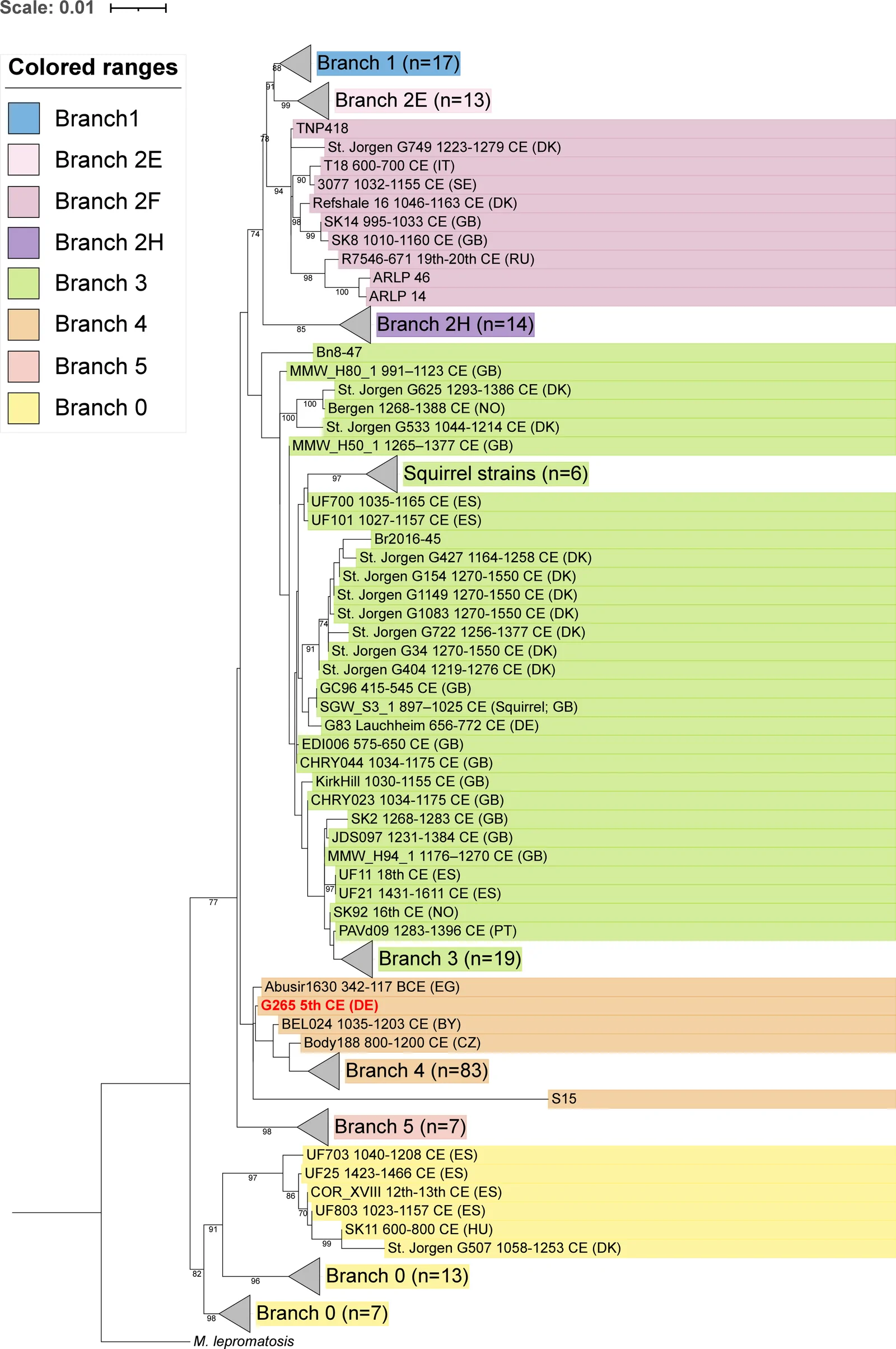
Maximum likelihood tree of *Mycobacterium leprae* based on 3,124 single nucleotide polymorphisms The tree is rooted using the outgroup *Mycobacterium lepromatosis*. Legend colors indicate the corresponding branch. Branches containing only modern strains have been collapsed. Ancient strains have the corresponding dates and abbreviated country names shown (list of abbreviations in Table S6). G265 is marked in red. Genomes used for constructing the phylogenetic tree are listed in Table S6. The scale bar represents the number of nucleotide substitutions per site. Bootstrap values greater than 70% are displayed numerically on branch nodes.

Next, we investigated known *M. leprae* virulence factors. We examined all *M. leprae-*specific genes listed in the Virulence Factor Database (VFDB).^35^ The G265 strain exhibited more than 95% coverage of all 160 genes (Table S5). In particular, it showed a high coverage of several genes involved in the type VII secretion system (including *ecc*, *esp*, and *esx* genes^36^^,^^37^; Table S5), the antigen 85 complex (*fbpA*, *fbpB*, and *fbpC*^37^^,^^38^; Table S5) and the production of phenolic glycolipids (*pks15/1*, *mas*, *fadD*, *pap*, and *ppsA-E*^39^^,^^40^^,^^41^; Table S5).

## Discussion

In the present study, we conducted metagenomic analyses on skeletal samples obtained from 11 individuals buried near the dwelling mound Fallward in northwestern Germany. The cemetery is known for its exceptionally well-preserved wooden and textile materials, rendering it one of the most important Roman Iron Age to Migration Period burial sites in central Europe.^42^ In contrast, the quality and quantity of the extracted human DNA was relatively poor. It was also noted that the *M. leprae* genome exhibited substantially better preservation than the host’s endogenous DNA. The number of bacterial reads exceeded human reads by a considerable margin, an observation that has been previously reported.^12^^,^^13^^,^^43^ This difference may be due to the mycobacterium’s lipid-rich cell wall, which could confer greater protection against postmortem degradation.^12^

For four individuals, the genetic results were consistent with the osteological sex determinations and aligned with the associated burial goods (Table 1; Figure S1). Two children, G282 and G224, showed discrepancies between their genetically inferred male sex and their gender suggested by the grave assemblages, which included brooches and beads often interpreted as female-associated attributes. These inconsistencies highlight the limitations of directly equating grave goods with biological sex, particularly in children, for whom gender roles and social identities may not have been rigidly defined or consistently expressed in burial practices. It is also possible that the grave goods may reflect familial, symbolic or ritual aspects rather than the biological sex of the individual. However, given the low genomic coverage and reduced damage signals for these individuals, their genetic sex assignments should be interpreted with caution.

The genomes of all three individuals for which sufficient data could be generated (G14, G250, and G265) clustered with contemporaneous European populations (Figure 3). In particular, G14 and G250, who fell within the variation observed in northwestern Europe today, were closely related to Anglo-Saxons (450–1066 CE; Figure 4). This finding could indicate that their relatives or descendants (in case of G14) might have contributed to the migration across the North Sea to the British Isles. In contrast, G265, an adult male estimated to be approximately 50 years old, clustered with Roman Iron Age and Migration Period individuals from frontier provinces along the Danubian region (Figure 4), suggesting genetic affinities to populations from eastern central Europe (Figure 5). His burial near Fallward suggests he or his recent ancestors may have originated somewhere southeast of the burial site, potentially within the broader Danubian frontier region, before migrating to Fallward. However, as complementary analyses lacked sufficient resolution to refine his exact genetic origin, this interpretation should be treated cautiously.

At the time of his death in the second quarter of the 5^th^ century CE,^4^ G265 was infected with *M. leprae*, though his skeletal remains did not show any leprosy-specific lesions. The reconstructed bacterial genome belongs to branch 4, whose earliest known representative dates to Egypt between the 4^th^ and 2^nd^ centuries BCE. By SNP effect analysis, we detected no unique high-impact SNPs. The G265 strain exhibited high coverage of all known *M. leprae* virulence factors. Leprosy is a chronic disease and can take years to develop.^44^ The apparent absence of bone pathologies may indicate that G265 was affected by an asymptomatic form of leprosy. Alternatively, it is possible that he showed symptoms in soft tissues without inflammation in the skeleton. In any case, he probably died from an unrelated secondary cause.

In addition to his infection with a branch-4 *M. leprae* strain, G265 stands out as a particularly noteworthy individual due to the distinctive nature of his grave goods. The man was buried in an oak (*Quercus*) boat over four meters long that was carefully sealed with a roof-like structure (Figure 2A). The boat suggests that during his lifetime he may have had a strong connection to the nearby rivers or the surrounding maritime environment. This interpretation is supported by the reed mat on which the body was laid, the presence of several fish bones found within the boat, and a small box shaped like a water bird. Osteological analysis further reveals that the man had particularly well-developed upper arm muscles. Taken together, these clues could point to frequent activity as a boatman or fisherman.^45^ Placed outside the boat was a block chair made of alder (*Alnus*), adorned with notched carvings and presumably painted (Figure 2B). The chair, often interpreted as a “throne”, is considered a clear symbol of authority or power. At the feet of the deceased was a maple (*Acer*) stool featuring a hunting scene and a runic inscription (Figure 2C). In addition, the grave contained a game table, a bronze basin and a ceramic vessel, among other items. The objects show clear signs of use and repair, indicating that none of them were made specifically for the burial. Instead, they likely belonged to the man himself or originated from his immediate personal or familial context, providing valuable insights into his life and daily routine.^45^

The quantity and quality of the objects are seen as reflecting the individual’s privileged status and material wealth within the local community.^4^ Although the results of the archeobotanical analysis suggest that only locally sourced wood was used,^4^ the varied and intricate decoration of the objects clearly reflect the influence of late antique art and craftsmanship.^42^ This connection to the Roman Empire is further underscored by the presence of a Roman military belt discovered in the grave, reinforcing the possibility that he or his ancestors originated from a region under Roman influence. Hypothetically, he might have been a *foederatus*—a mercenary who served in the Roman army and later sought a new home in the northwest of the continent.^8^ However, the belt itself does not necessarily imply Roman military service as such objects could also have been acquired through trade, inheritance or as a spoil of war.

In summary, the combined archeological and human/pathogen genomic findings from the Fallward burials highlight the complexity of individual biographies. Although the precise origin of G265 remains unresolved, the available evidence raises the possibility of long-distance connections involving northwestern Germany during the Late Roman and Migration periods.

### Limitations of the study

Several samples exhibited low genomic coverage and reduced terminal deamination signals (Table S1), reducing confidence in some analyses. This is particularly relevant for G224 and G282, where genetic sex assignments differ from osteological or archeological assessments and should therefore be interpreted with caution. For population genetic analyses, only three individuals (G14, G250, and G265) yielded genomic data of sufficient quality, although SNP coverage on the 1240K panel remained relatively low (32,000–67,000 covered SNPs; Table S1). This limited the resolution of comparative analyses, particularly those aimed at assessing the ancestry and possible geographic origin of G265. Accordingly, qpAdm modeling did not support a distinct ancestry model for G265 relative to the other two Fallward individuals, as several one-way models based on northern and central European Iron Age and Migration Period populations could not be rejected for any of the three individuals, while two-way models were generally infeasible (Table S2). This likely reflects the close genetic similarity among candidate source populations and the limited resolution of the available outgroups, further compounded by the low SNP coverage of the Fallward genomes. G14 and G265 exhibited lower terminal C to T misincorporation levels than G250 (Figure S6). Although substantial variation in deamination levels is expected among ancient samples and no universally accepted threshold for postmortem damage has been established, the combination of relatively low genomic coverage and reduced damage patterns warrants cautious interpretation of the presented population genetic results. Finally, because these analyses are based on only three individuals, the findings should be regarded as individual case studies and may not reflect the genetic diversity of the broader Fallward community.

## Resource availability

### Lead contact

Requests for further information and resources should be directed to and will be fulfilled by the lead contact, Ben Krause-Kyora (b.krause-kyora@ikmb.uni-kiel.de).

### Materials availability

This study did not generate new unique reagents. The skeletal material analyzed in this study is held by the responsible curating institutions. Access to the specimens was granted by curating institutions and associated researchers, including co-authors S.G.-P. (Ossatura, Braunschweig), H.J., and C.P. (Niedersächsisches Institut für historische Küstenforschung, Wilhelmshaven). Researchers seeking access to these collections should contact the relevant curating institutions and co-authors directly.

### Data and code availability

• Raw sequencing data generated in this study have been deposited in the European Nucleotide Archive (ENA: PRJEB101719) and are publicly available as of the date of publication.
• This paper does not report original code.
• Any additional information required to reanalyze the data reported in this paper is available from the lead contact upon request.

## Acknowledgments

This study was funded by the 10.13039/501100001659Deutsche Forschungsgemeinschaft (DFG, German Research Foundation) under Germany’s Excellence Strategy – ROOTS EXC 2150 390870439. D.A.M. is supported by a doctoral scholarship awarded by the Federal State of Schleswig-Holstein, Germany.

## Author contributions

Conceptualization, A.N. and B.K.-K.; data curation, D.A.M. and N.A.d.S.; formal analysis, D.A.M., N.A.d.S., and J.H.R.-D.; funding acquisition, B.K.-K.; investigation, D.A.M., N.A.d.S., C.P., H.J., A.N., and B.K.-K.; project administration, A.N. and B.K.-K.; resources, S.G.-P., H.J., and C.P.; supervision, A.N. and B.K.-K.; visualization, D.A.M. and N.A.d.S.; writing – original draft, A.N., D.A.M., N.A.d.S., C.P., and B.K.-K.; writing – review and editing, D.A.M., N.A.d.S., J.H.R.-D., S.G.-P., H.J., C.P., A.N., and B.K.-K. All authors reviewed the paper and approved the decision to submit the paper.

## Declaration of interests

The authors declare no competing interests.

## STAR★Methods

### Key resources table

**Table:** 

REAGENT or RESOURCE	SOURCE	IDENTIFIER
**Biological samples**		

Human archaeological remains	This study	G14
Human archaeological remains	This study	G223
Human archaeological remains	This study	G224
Human archaeological remains	This study	G250
Human archaeological remains	This study	G265
Human archaeological remains	This study	G282
Human archaeological remains	This study	G284
Human archaeological remains	This study	G407
Human archaeological remains	This study	G439
Human archaeological remains	This study	G452
Human archaeological remains	This study	G468

**Deposited data**		

Sequencing data	This study	ENA: PRJEB101719

**Software and algorithms**		

Clip&Merge v1.7.8	Peltzer et al.^46^	https://github.com/apeltzer/ClipAndMerge
BWA v0.7.15	Li and Durbin,^47^	https://github.com/lh3/bwa
DeDup v0.12.1	Peltzer et al.^46^	https://github.com/apeltzer/DeDup
bamUtil v1.0.15	Jun et al.^48^	https://github.com/statgen/bamUtil
SAMtools v1.12	Li et al.^49^	https://www.htslib.org/
DamageProfiler v.1.1	Neukamm et al.^50^	https://github.com/Integrative-Transcriptomics/DamageProfiler
Schmutzi v1.5.5.5	Renaud et al.^51^	https://github.com/grenaud/schmutzi
ANGSD v0.935	Korneliussen et al.^52^	https://github.com/ANGSD/angsd
HapCon v0.64	Huang and Ringbauer,^53^	https://github.com/hringbauer/hapROH
HaploGrep2 v2.4.0	Weissensteiner et al.^54^	https://github.com/seppinho/haplogrep-cmd
yHaplo v1.1.2	Poznik,^55^	https://github.com/23andMe/yhaplo
SequenceTools v1.2.2	Schiffels and Peltzer, 2023	https://github.com/stschiff/sequenceTools
Allen Ancient DNA Resource (AADR), version 62.0	Mallick et al.^56^	https://dataverse.harvard.edu/dataset.xhtml?persistentId=doi:10.7910/DVN/FFIDCW
smartpca v16000	Patterson et al.^57^	https://github.com/DReichLab/EIG
qp3Pop v650	Patterson,^58^	https://github.com/DReichLab/AdmixTools
qpDstat v970	Patterson et al.^58^	https://github.com/DReichLab/AdmixTools
ADMIXTURE v1.3.0	Alexander et al.^59^	https://dalexander.github.io/admixture/
PLINK v1.90b6.21	Purcell et al.^60^	https://www.cog-genomics.org/plink/
qpAdm v1201	Patterson et al.^58^	https://github.com/DReichLab/AdmixTools
mobest v1.0.0	Schmid & Schiffels,^61^	https://github.com/nevrome/mobest
MALT v0.4.1	Vågene et al.^62^	https://uni-tuebingen.de/fakultaeten/mathematisch-naturwissenschaftliche-fakultaet/fachbereiche/informatik/lehrstuehle/algorithms-in-bioinformatics/software/malt/
MEGAN6 v6.25.9	Huson et al.^63^	https://uni-tuebingen.de/fakultaeten/mathematisch-naturwissenschaftliche-fakultaet/fachbereiche/informatik/lehrstuehle/algorithms-in-bioinformatics/software/megan6/
IGV v2.17.1	Robinson et al.^64^	https://igv.org/
BLAST v2.15.0	Altschul et al.^65^	https://blast.ncbi.nlm.nih.gov/Blast.cgi?PROGRAM=blastn&PAGE_TYPE=BlastSearch&LINK_LOC=blasthome
BCFtools v1.12	Li,^66^	https://www.htslib.org/
filterSAM v0.0.11	Robaina-Estévez,^67^	https://github.com/Robaina/filterSAM
RAxML v8.2.12	Stamatakis et al.^68^	https://cme.h-its.org/exelixis/web/software/raxml/
iTOL v6	Letunic and Bork,^69^	https://itol.embl.de/
SnpEff v5.2f	Cingolani et al.^70^	https://pcingola.github.io/SnpEff/
VFDB	Liu et al.^35^	https://www.mgc.ac.cn/VFs/

### Experimental model and study participant details

#### Archeological context

This study was conducted in full compliance with the ethical principles of DNA research on historical human remains.^71^ No genomic data was generated from living individuals or other modern human remains.

As part of anthropological investigations,^9^ 12 samples were collected from the skeletal remains of 11 individuals whose inhumation burials had been excavated between 1995 and 1998. Since all graves represent single interments, the skeletal remains could be clearly attributed to specific individuals.

The deceased had been interred, at depths of up to two meters, in their clothing and accompanied by numerous grave goods made of a wide variety of organic and inorganic materials. The exceptionally well-preserved grave goods indicate that the burials date to the 4^th^ and 5^th^ centuries CE. Several graves were examined and documented directly in the field (G223, G224, G439, G452, G265, G468), while others were recovered as intact blocks and subsequently excavated under laboratory conditions (G14, G250, G282, G284, G407).

Many of the grave goods were conserved according to their respective materials and are now on museum display. The human skeletal remains, however, remained in storage until the recent anthropological analyses were conducted. At present, the burial site at Fallward is being re-examined and evaluated within the framework of an interdisciplinary research project funded by the German Research Foundation (DFG).^4^^,^^7^

### Method details

#### Sample processing and sequencing

Following published guidelines,^72^^,^^73^ all laboratory work was carried out in the clean rooms of the Ancient DNA Laboratory at Kiel University. Surfaces were thoroughly cleaned with sodium hypochlorite and/or nucleic acid removal reagents before and after use. UV sterilization was also used to disinfect all workspaces. Throughout the experiments, laboratory staff wore coveralls, gloves and face masks. All chemicals and reagents were free of DNases, RNases and pyrogens, and only filtered pipette tips were employed. The 12 samples were prepared and powdered as described earlier.^43^ Lastly, DNA was extracted using previously established methods.^43^^,^^74^

Using an approach outlined in previous studies,^43^^,^^75^ double-stranded, half-UDG treated libraries were generated. These libraries were shotgun sequenced at the Institute of Clinical Molecular Biology at Kiel University, using the Illumina NovaSeq 6000 (2 × 100 bp) platform. Raw reads were then processed to remove adaptors and forward and reverse sequences merged using Clip&Merge v1.7.8^46^ with default settings, which includes a filter to retain reads with at least 25bp.

### Quantification and statistical analysis

#### Mapping to human reference genome

Sequencing reads were aligned to the hg19 human reference genome using Burrows-Wheeler Aligner (BWA) aln v0.7.15^47^ with parameters optimised for ancient DNA (relaxed mismatch: -n 0.01, disabled seeding: -l 300). PCR duplicates were removed using DeDup v0.12.1.^46^ Unless otherwise stated, all downstream analyses were performed on reads with the last two terminal bases trimmed with bamUtil v1.0.15^48^ and filtered to a minimum base and mapping quality of 30 using SAMtools v1.12.^49^ BAM files that did not undergo trimming and mapping quality filtering steps are used for some analyses and are referred to as “unfiltered” in the subsequent sections. Mapping statistics are displayed in Table S1.

#### Ancient DNA authentication

Postmortem damage patterns were assessed with DamageProfiler v1.1^50^ and contamination levels were estimated from mitochondrial DNA (mtDNA) using Schmutzi v1.5.5.5,^51^ both on unfiltered BAM files. Contamination from the X chromosome in male individuals was assessed using both ANGSD v0.935^52^ and HapCon^53^ from the HapROH package v0.64.^76^ For HapCon, the 1000 Genomes reference panel was used and estimates based on fewer than 1,000 target sites were considered unreliable and therefore excluded. Deamination plots are displayed in Figure S6. Contamination estimates are presented in Table S1. For sample G265, sequencing data was available from four libraries derived from two skeletal elements (calcaneus bone and tooth). Contamination estimates obtained with HapCon were elevated when all libraries were merged. Therefore, only a single library, the best-covered dataset from the calcaneus bone, was retained for downstream human population genetic analyses, as it appeared uncontaminated when assessed independently. However, for subsequent pathogen analysis, all four libraries were used, as their deamination patterns when mapped against *M. leprae* were consistent with ancient DNA. Moreover, human contamination is highly unlikely to explain the detection of *M. leprae* and is unlikely to influence downstream analyses of *M. leprae*.

#### Genetic sex determination and uniparental lineage assignment

Biological sex was inferred using two metrics comparing sequence read depth on the sex chromosomes to that on the autosomes.^77^^,^^78^ Genetic sex results are shown in Figure S1. Mitochondrial haplogroups were determined with HaploGrep2 v2.4.0,^54^ while Y chromosome haplogroups for male individuals were identified using yHaplo v1.1.2.^55^ Mitochondrial and Y chromosome haplogroups are presented in Table S1.

#### Genotyping and merging to publicly available data

Pseudo-haploid genotype calls were generated at single SNP sites included in the 1240K and Human Origins (HO) panels with SequenceTools v1.2.2 (https://github.com/stschiff/sequenceTools). Individuals represented by fewer than 20,000 covered SNPs on the 1240K panel were excluded from downstream population genetic analyses. The remaining data from three individuals (G14, G250 and G265) were merged with reference datasets from the Allen Ancient DNA Resource (AADR), version 62.0,^56^ for both SNP panels. Additionally, published data relevant to the study region and period, but not yet included in the AADR, were incorporated into the reference dataset.^79^^,^^80^^,^^81^^,^^82^^,^^83^^,^^84^^,^^85^ Genotyping statistics are shown in Table S1.

#### Population structure and ancestry modeling

Principal component analysis (PCA) was carried out using smartpca v16000,^57^ with the “lsqproject: YES” option and projecting ancient individuals onto the variation observed in present-day West-Eurasian populations from the HO reference dataset. PCA results are displayed in Figures 3 and 4. Outgroup f_3_ statistics and f_4_ statistics were computed with qp3Pop v650^58^ and qpDstat v970,^58^ respectively. Both analyses were performed using the 1240K dataset and employing Mbuti as the outgroup. f_3_ statistics and f_4_ statistics are presented in Figures S2 and S3. To explore ancestry components, we performed unsupervised clustering with ADMIXTURE v1.3.0,^59^ testing values of K between 3 and 12 and using 200 bootstrap replicates for cross-validation. The analysis used a linkage-disequilibrium–pruned HO dataset generated in PLINK v1.90b6.21^60^ with the parameters --indep-pairwise 200 25 0.4. ADMIXTURE results are shown in Figure S4. Admixture modeling was additionally done with qpAdm v1201^58^ using the parameter “allsnps: YES”. The set of right populations used for qpAdm analysis along with the results are listed in Table S2.

#### Geographic origin inference for individual G265

To model the likely geographic origin of G265, we employed mobest v1.0.0,^61^ a tool that performs spatiotemporal interpolation of genetic ancestry using Gaussian process regression and estimates the probability of genetic similarity across geographic space. The first three principal components from the PCA described above were used as ancestry coordinates. The reference dataset comprised 5,598 ancient individuals that passed AADR quality assessment (PASS), had >20,000 covered 1240K SNPs, were dated between 1200 BCE and 2000 CE, and were located within Europe and the Mediterranean region. Samples annotated in the AADR metadata as duplicates, low quality, contaminated, or close relatives were excluded, and only one individual per Master ID was retained. Geographic coordinates were transformed to EPSG3035 and ancestry fields were interpolated on a 25 km prediction grid covering the study region. Gaussian process kernels were parameterized using the default settings of mobest (spatial length scale = 800 km, temporal length scale = 800 years, nugget = 0.1 for each principal component). The geographic origin of G265 was inferred by evaluating the interpolated ancestry field around 500 CE, corresponding approximately to his archeological date. Geographic origin inference results are presented in Figure 5.

#### Pathogen screening

The Megan Alignment Tool (MALT) v0.4.1^62^ was used to align the processed reads against a curated reference database consisting of 27,730 bacterial and 10,543 viral complete genomes downloaded from NCBI.^86^ MALT parameters were set to BlastN mode with semi-global alignment and a minimum percent identity of 95%. MALT alignments were then inspected using the Metagenome Analyzer 6 (MEGAN6) v6.25.9.^63^ Subsequent mapping was carried out against bacterial or viral references using the BWA aln^47^ algorithm (parameters described above). Duplicate reads were removed with DeDup v0.12.1^46^ and deamination plots were created using DamageProfiler v.1.1.^50^ Deamination plots for *M. leprae* are shown in Figure S5. Mapping statistics were generated by SAMtools v1.12^49^ (*M. leprae* mapping statistics are reported in Table S3). Read alignments were manually inspected using Integrative Genomics Viewer (IGV) v2.17.1^64^ to assess coverage uniformity and depth as well as read quality and the average number of SNPs per read. Finally, aligned reads were analyzed with the online version of the Basic Local Alignment Search Tool (BLAST) v2.15.0^65^ to determine whether the expect-value (E-value) was significant (less than 1 × 10^−5^).

#### *Mycobacterium leprae* phylogenetics

Modern and ancient *M. leprae* genomes were downloaded from the NCBI and European Nucleotide Archive (ENA) databases (Table S6). Genomes downloaded in FASTA format were converted to FASTQ with the wgsim tool from SAMtools v1.12,^49^ with the rate of mutations, fraction of indels, the probability that an indel is extended and base error rate all set to zero. Using the same BWA aln^47^ parameters described above, all FASTQ files were then mapped against the reference genome *M. leprae* strain TN (NCBI RefSeq: NC_002677.1). Generated BAM files were converted to VCF format using BCFtools v1.12^66^ with the following parameters: read depth of 3, indelgap set to 5 and trimming of 2 positions. Additionally, SAMtools v1.12^49^ was used to filter out reads with quality below 30 and a percent identity filter of 95% was applied using filterSAM v0.0.11.^67^ VCFs were subsequently filtered by 3,124 positions which were previously described as informative for *M. leprae* phylogenetics.^15^ The phylogenetic tree was generated using RAxML v8.2.12^68^ with the following parameters: -f a -x 12345 -p 12345 -# 1000 -N 1000 -m GTRGAMMA. Annotations were made via the online Interactive tree of life (iTOL) v6.^69^ The phylogeny is presented in Figure 6.

#### Single nucleotide polymorphism effect analysis

In order to carry out SNP effect analysis, 44 ancient and nine representative modern strains used in the phylogeny were selected. BAM files for these 53 strains and the G265 strain were converted to VCF format, as described above for the *M*. *leprae* phylogenetics. However, VCFs were not filtered by the 3,124 informative positions. SnpEff v5.2f^70^ was then used to annotate these VCFs and classify the SNP impact. The output generated by SnpEff was parsed to display only variants annotated with “HIGH” impact which were found in the G265 strain (Table S4).

#### Virulence factor analysis

All *M. leprae* specific virulence genes (*n* = 160) were downloaded from the Virulence Factor Database (VFDB)^35^. Thereafter, reads generated from G265 were mapped against these genes with BWA aln^47^ using abovementioned parameters. BAM files were then filtered by a mapping quality of 30 using SAMtools v1.12^49^ and a percent identity filter of 95% was applied using filterSAM v0.0.11.^67^ Mapping statistics for each gene were summarized using SAMtools v1.12^49^ and compiled into a tabular format (Table S5).

## Supplemental information

Document S1. Figures S1–S6

Table S1. Summary from the population genetic analyses

Table S2. Results from qpAdm modeling

Table S3. Mapping statistics for reads generated from the individual in G265 mapped to *Mycobacterium leprae* TN after duplicate removal

Table S4. SnpEff analysis results showing only high impact variants

Table S5. Coverage of *Mycobacterium leprae* virulence factors after filtering by mapping quality 30 and percent identity 95

Table S6. *Mycobacterium leprae* reference genomes

## References

[1] Struckmeyer K., Jöns H. (2020).

[2] Behre K.-E., Haarnagel W., Kossack G., Behre K.-E., Schmid P. (1984). Archäologische und naturwissenschaftliche Untersuchungen an ländlichen und frühstädtischen Siedlungen im deutschen Küstengebiet vom 5. Jahrhundert v. Chr. bis zum 11. Jahrhundert nach Chr. Band 1: Ländliche Siedlungen.

[3] Aufderhaar I. (2016). Sievern, Ldkr. Cuxhaven – Analyse einer Zentralregion von der ausgehenden Vorrömischen Eisenzeit bis in das 6. Jh. n. Chr.

[4] Peek C., Jöns H., Behre K.-E., Schön M.D. (2021).

[5] Behr C., Hines J., Ludowici B. (2020). Saxones. Neue Studien zur Sachsenforschung.

[6] Gretzinger J., Sayer D., Justeau P., Altena E., Pala M., Dulias K., Edwards C.J., Jodoin S., Lacher L., Sabin S. (2022). The Anglo-Saxon migration and the formation of the early English gene pool. Nature.

[7] Peek C. (2025).

[8] Schön M.D. (2010). Studien zur Landschafts- und Siedlungsgeschichte im südlichen Nordseegebiet.

[9] Grefen-Peters S. (2021).

[10] Åhlfeldt J. (2020). Digital Atlas of the Roman Empire. https://imperium.ahlfeldt.se/.

[11] Rootsi S., Myres N.M., Lin A.A., Järve M., King R.J., Kutuev I., Cabrera V.M., Khusnutdinova E.K., Varendi K., Sahakyan H. (2012). Distinguishing the co-ancestries of haplogroup G Y-chromosomes in the populations of Europe and the Caucasus. Eur. J. Hum. Genet..

[12] Schuenemann V.J., Singh P., Mendum T.A., Krause-Kyora B., Jäger G., Bos K.I., Herbig A., Economou C., Benjak A., Busso P. (2013). Genome-wide comparison of medieval and modern Mycobacterium leprae. Science.

[13] Neukamm J., Pfrengle S., Molak M., Seitz A., Francken M., Eppenberger P., Avanzi C., Reiter E., Urban C., Welte B. (2020). 2000-year-old pathogen genomes reconstructed from metagenomic analysis of Egyptian mummified individuals. BMC Biol..

[14] Pfrengle S., Neukamm J., Guellil M., Keller M., Molak M., Avanzi C., Kushniarevich A., Montes N., Neumann G.U., Reiter E. (2021). Mycobacterium leprae diversity and population dynamics in medieval Europe from novel ancient genomes. BMC Biol..

[15] Schuenemann V.J., Avanzi C., Krause-Kyora B., Seitz A., Herbig A., Inskip S., Bonazzi M., Reiter E., Urban C., Dangvard Pedersen D. (2018). Ancient genomes reveal a high diversity of Mycobacterium leprae in medieval Europe. PLoS Pathog..

[16] Inskip S.A., Taylor G.M., Zakrzewski S.R., Mays S.A., Pike A.W.G., Llewellyn G., Williams C.M., Lee O.Y.-C., Wu H.H.T., Minnikin D.E. (2015). Osteological, Biomolecular and Geochemical Examination of an Early Anglo-Saxon Case of Lepromatous Leprosy. PLoS One.

[17] Bonczarowska J.H., Susat J., Mühlemann B., Jasch-Boley I., Brather S., Höke B., Brather-Walter S., Schoenenberg V., Scheschkewitz J., Graenert G. (2022). Pathogen genomics study of an early medieval community in Germany reveals extensive co-infections. Genome Biol..

[18] Urban C., Blom A.A., Avanzi C., Walker-Meikle K., Warren A.K., White-Iribhogbe K., Turle R., Marter P., Dawson-Hobbis H., Roffey S. (2024). Ancient *Mycobacterium leprae* genome reveals medieval English red squirrels as animal leprosy host. Curr. Biol..

[19] Taylor G.M., Tucker K., Butler R., Pike A.W.G., Lewis J., Roffey S., Marter P., Lee O.Y.-C., Wu H.H.T., Minnikin D.E. (2013). Detection and Strain Typing of Ancient Mycobacterium leprae from a Medieval Leprosy Hospital. PLoS One.

[20] Fotakis A.K., Denham S.D., Mackie M., Orbegozo M.I., Mylopotamitaki D., Gopalakrishnan S., Sicheritz-Pontén T., Olsen J.V., Cappellini E., Zhang G. (2020). Multi-omic detection of Mycobacterium leprae in archaeological human dental calculus. Philos. Trans. R. Soc. Lond. B Biol. Sci..

[21] Stefani M.M.A., Avanzi C., Bührer-Sékula S., Benjak A., Loiseau C., Singh P., Pontes M.A.A., Gonçalves H.S., Hungria E.M., Busso P. (2017). Whole genome sequencing distinguishes between relapse and reinfection in recurrent leprosy cases. PLoS Negl. Trop. Dis..

[22] Romero-Alvarez D., Garzon-Chavez D., Jackson M., Avanzi C., Peterson A.T. (2023). Mycobacterium leprae in Armadillo Tissues from Museum Collections, United States. Emerg. Infect. Dis..

[23] Hockings K.J., Mubemba B., Avanzi C., Pleh K., Düx A., Bersacola E., Bessa J., Ramon M., Metzger S., Patrono L.V. (2021). Leprosy in wild chimpanzees. Nature.

[24] Benjak A., Avanzi C., Singh P., Loiseau C., Girma S., Busso P., Fontes A.N.B., Miyamoto Y., Namisato M., Bobosha K. (2018). Phylogenomics and antimicrobial resistance of the leprosy bacillus Mycobacterium leprae. Nat. Commun..

[25] Avanzi C., del-Pozo J., Benjak A., Stevenson K., Simpson V.R., Busso P., McLuckie J., Loiseau C., Lawton C., Schoening J. (2016). Red squirrels in the British Isles are infected with leprosy bacilli. Science.

[26] Avanzi C., Busso P., Benjak A., Loiseau C., Fomba A., Doumbia G., Camara I., Lamou A., Sock G., Drame T. (2016). Transmission of Drug-Resistant Leprosy in Guinea-Conakry Detected Using Molecular Epidemiological Approaches. Clin. Infect. Dis..

[27] Mendum T.A., Schuenemann V.J., Roffey S., Taylor G.M., Wu H., Singh P., Tucker K., Hinds J., Cole S.T., Kierzek A.M. (2014). Mycobacterium leprae genomes from a British medieval leprosy hospital: towards understanding an ancient epidemic. BMC Genom..

[28] Truman R.W., Singh P., Sharma R., Busso P., Rougemont J., Paniz-Mondolfi A., Kapopoulou A., Brisse S., Scollard D.M., Gillis T.P., Cole S.T. (2011). Probable Zoonotic Leprosy in the Southern United States. N. Engl. J. Med..

[29] Singh P., Benjak A., Schuenemann V.J., Herbig A., Avanzi C., Busso P., Nieselt K., Krause J., Vera-Cabrera L., Cole S.T. (2015). Insight into the evolution and origin of leprosy bacilli from the genome sequence of Mycobacterium lepromatosis. Proc. Natl. Acad. Sci..

[30] Singh P., Benjak A., Carat S., Kai M., Busso P., Avanzi C., Paniz-Mondolfi A., Peter C., Harshman K., Rougemont J. (2014). Genome-wide re-sequencing of multidrug-resistant Mycobacterium leprae Airaku-3. Clin. Microbiol. Infect..

[31] Honap T.P., Pfister L.-A., Housman G., Mills S., Tarara R.P., Suzuki K., Cuozzo F.P., Sauther M.L., Rosenberg M.S., Stone A.C. (2018). Mycobacterium leprae genomes from naturally infected nonhuman primates. PLoS Negl. Trop. Dis..

[32] Sharma R., Singh P., Loughry W.J., Lockhart J.M., Inman W.B., Duthie M.S., Pena M.T., Marcos L.A., Scollard D.M., Cole S.T., Truman R.W. (2015). Zoonotic Leprosy in the Southeastern United States - Volume 21. Emerg. Infect. Dis..

[33] Blevins K.E., Crane A.E., Lum C., Furuta K., Fox K., Stone A.C. (2020). Evolutionary history of Mycobacterium leprae in the Pacific Islands. Philos. Trans. R. Soc. Lond. B Biol. Sci..

[34] Monot M., Honoré N., Garnier T., Zidane N., Sherafi D., Paniz-Mondolfi A., Matsuoka M., Taylor G.M., Donoghue H.D., Bouwman A. (2009). Comparative genomic and phylogeographic analysis of Mycobacterium leprae. Nat. Genet..

[35] Liu B., Zheng D., Zhou S., Chen L., Yang J. (2022). VFDB 2022: a general classification scheme for bacterial virulence factors. Nucleic Acids Res..

[36] Abdallah A.M., Gey van Pittius N.C., Champion P.A.D., Cox J., Luirink J., Vandenbroucke-Grauls C.M.J.E., Appelmelk B.J., Bitter W. (2007). Type VII secretion — mycobacteria show the way. Nat. Rev. Microbiol..

[37] Cole S.T., Eiglmeier K., Parkhill J., James K.D., Thomson N.R., Wheeler P.R., Honoré N., Garnier T., Churcher C., Harris D. (2001). Massive gene decay in the leprosy bacillus. Nature.

[38] Pessolani M.C., Brennan P.J. (1992). Mycobacterium leprae produces extracellular homologs of the antigen 85 complex. Infect. Immun..

[39] Hunter S.W., Brennan P.J. (1981). A novel phenolic glycolipid from Mycobacterium leprae possibly involved in immunogenicity and pathogenicity. J. Bacteriol..

[40] Trivedi O.A., Arora P., Sridharan V., Tickoo R., Mohanty D., Gokhale R.S. (2004). Enzymic activation and transfer of fatty acids as acyl-adenylates in mycobacteria. Nature.

[41] Girardi K.d.C.d.V., Mietto B.S., Dos Anjos Lima K., Atella G.C., da Silva D.S., Pereira A.M.R., Rosa P.S., Lara F.A. (2023). Phenolic glycolipid-1 of Mycobacterium leprae is involved in human Schwann cell line ST8814 neurotoxic phenotype. J. Neurochem..

[42] Schön M.D. (2006). Reallexikon der Germanischen Altertumskunde (RGA).

[43] Krause-Kyora B., Nutsua M., Boehme L., Pierini F., Pedersen D.D., Kornell S.-C., Drichel D., Bonazzi M., Möbus L., Tarp P. (2018). Ancient DNA study reveals HLA susceptibility locus for leprosy in medieval Europeans. Nat. Commun..

[44] White C., Franco-Paredes C. (2015). Leprosy in the 21st Century. Clin. Microbiol. Rev..

[45] Peek C., Hüser A., Meier U.M. (2022). Die Gräber der Fallward. Aus­ stellung im Museum Burg Bederkesa.

[46] Peltzer A., Jäger G., Herbig A., Seitz A., Kniep C., Krause J., Nieselt K. (2016). EAGER: efficient ancient genome reconstruction. Genome Biol..

[47] Li H., Durbin R. (2009). Fast and accurate short read alignment with Burrows-Wheeler transform. Bioinformatics.

[48] Jun G., Wing M.K., Abecasis G.R., Kang H.M. (2015). An efficient and scalable analysis framework for variant extraction and refinement from population-scale DNA sequence data. Genome Res..

[49] Li H., Handsaker B., Wysoker A., Fennell T., Ruan J., Homer N., Marth G., Abecasis G., Durbin R., 1000 Genome Project Data Processing Subgroup (2009). The Sequence Alignment/Map format and SAMtools. Bioinformatics.

[50] Neukamm J., Peltzer A., Nieselt K. (2021). DamageProfiler: fast damage pattern calculation for ancient DNA. Bioinformatics.

[51] Renaud G., Slon V., Duggan A.T., Kelso J. (2015). Schmutzi: estimation of contamination and endogenous mitochondrial consensus calling for ancient DNA. Genome Biol..

[52] Korneliussen T.S., Albrechtsen A., Nielsen R. (2014). ANGSD: Analysis of Next Generation Sequencing Data. BMC Bioinf..

[53] Huang Y., Ringbauer H. (2022). hapCon: estimating contamination of ancient genomes by copying from reference haplotypes. Bioinformatics.

[54] Weissensteiner H., Pacher D., Kloss-Brandstätter A., Forer L., Specht G., Bandelt H.-J., Kronenberg F., Salas A., Schönherr S. (2016). HaploGrep 2: mitochondrial haplogroup classification in the era of high-throughput sequencing. Nucleic Acids Res..

[55] Poznik G.D. (2016). Identifying Y-chromosome haplogroups in arbitrarily large samples of sequenced or genotyped men. bioRxiv.

[56] Mallick S., Micco A., Mah M., Ringbauer H., Lazaridis I., Olalde I., Patterson N., Reich D. (2024). The Allen Ancient DNA Resource (AADR) a curated compendium of ancient human genomes. Sci. Data.

[57] Patterson N., Price A.L., Reich D. (2006). Population Structure and Eigenanalysis. PLoS Genet..

[58] Patterson N., Moorjani P., Luo Y., Mallick S., Rohland N., Zhan Y., Genschoreck T., Webster T., Reich D. (2012). Ancient admixture in human history. Genetics.

[59] Alexander D.H., Novembre J., Lange K. (2009). Fast model-based estimation of ancestry in unrelated individuals. Genome Res..

[60] Purcell S., Neale B., Todd-Brown K., Thomas L., Ferreira M.A.R., Bender D., Maller J., Sklar P., de Bakker P.I.W., Daly M.J., Sham P.C. (2007). PLINK: a tool set for whole-genome association and population-based linkage analyses. Am. J. Hum. Genet..

[61] Schmid C., Schiffels S. (2023). Estimating human mobility in Holocene Western Eurasia with large-scale ancient genomic data. Proc. Natl. Acad. Sci..

[62] Vågene Å.J., Herbig A., Campana M.G., Robles García N.M., Warinner C., Sabin S., Spyrou M.A., Andrades Valtueña A., Huson D., Tuross N. (2018). Salmonella enterica genomes from victims of a major sixteenth-century epidemic in Mexico. Nat. Ecol. Evol..

[63] Huson D.H., Beier S., Flade I., Górska A., El-Hadidi M., Mitra S., Ruscheweyh H.-J., Tappu R. (2016). MEGAN Community Edition - Interactive Exploration and Analysis of Large-Scale Microbiome Sequencing Data. PLoS Comput. Biol..

[64] Robinson J.T., Thorvaldsdóttir H., Winckler W., Guttman M., Lander E.S., Getz G., Mesirov J.P. (2011). Integrative genomics viewer. Nat. Biotechnol..

[65] Altschul S.F., Gish W., Miller W., Myers E.W., Lipman D.J. (1990). Basic local alignment search tool. J. Mol. Biol..

[66] Li H. (2011). A statistical framework for SNP calling, mutation discovery, association mapping and population genetical parameter estimation from sequencing data. Bioinformatics.

[67] Robaina-Estévez S. (2022).

[68] Stamatakis A. (2014). RAxML version 8: a tool for phylogenetic analysis and post-analysis of large phylogenies. Bioinformatics.

[69] Letunic I., Bork P. (2021). Interactive Tree Of Life (iTOL) v5: an online tool for phylogenetic tree display and annotation. Nucleic Acids Res..

[70] Cingolani P., Platts A., Wang L.L., Coon M., Nguyen T., Wang L., Land S.J., Lu X., Ruden D.M. (2012). A program for annotating and predicting the effects of single nucleotide polymorphisms, SnpEff. Fly (Austin).

[71] Alpaslan-Roodenberg S., Anthony D., Babiker H., Bánffy E., Booth T., Capone P., Deshpande-Mukherjee A., Eisenmann S., Fehren-Schmitz L., Frachetti M. (2021). Ethics of DNA research on human remains: five globally applicable guidelines. Nature.

[72] Fulton T.L., Shapiro B., Shapiro B., Barlow A., Heintzman P.D., Hofreiter M., Paijmans J.L.A., Soares A.E.R. (2019). Ancient DNA: Methods and Protocols.

[73] Cooper A., Poinar H.N. (2000). Ancient DNA: do it right or not at all. Science.

[74] Dabney J., Knapp M., Glocke I., Gansauge M.-T., Weihmann A., Nickel B., Valdiosera C., García N., Pääbo S., Arsuaga J.-L., Meyer M. (2013). Complete mitochondrial genome sequence of a Middle Pleistocene cave bear reconstructed from ultrashort DNA fragments. Proc. Natl. Acad. Sci. USA.

[75] Rohland N., Harney E., Mallick S., Nordenfelt S., Reich D. (2015). Partial uracil-DNA-glycosylase treatment for screening of ancient DNA. Philos. Trans. R. Soc. Lond. B Biol. Sci..

[76] Ringbauer H., Novembre J., Steinrücken M. (2021). Parental relatedness through time revealed by runs of homozygosity in ancient DNA. Nat. Commun..

[77] Skoglund P., Storå J., Götherström A., Jakobsson M. (2013). Accurate sex identification of ancient human remains using DNA shotgun sequencing. J. Archaeol. Sci..

[78] Mittnik A., Wang C.-C., Svoboda J., Krause J. (2016). A Molecular Approach to the Sexing of the Triple Burial at the Upper Paleolithic Site of Dolní Věstonice. PLoS One.

[79] Ravasini F., Conati Barbaro C., Scheib C.L., Tambets K., Metspalu M., Cruciani F., Trombetta B., D’Atanasio E. (2025). A New Perspective on the Arrival of the Eastern Mediterranean Genetic Influx in Central Italy Before the Onset of the Roman Empire. Genome Biol. Evol..

[80] Pilli E., Vai S., Moses V.C., Morelli S., Lari M., Modi A., Diroma M.A., Amoretti V., Zuchtriegel G., Osanna M. (2024). Ancient DNA challenges prevailing interpretations of the Pompeii plaster casts. Curr. Biol..

[81] Tian Y., Koncz I., Defant S., Giostra C., Vyas D.N., Sołtysiak A., Pejrani Baricco L., Fetner R., Posth C., Brandt G. (2024). The role of emerging elites in the formation and development of communities after the fall of the Roman Empire. Proc. Natl. Acad. Sci..

[82] Gretzinger J., Biermann F., Mager H., King B., Zlámalová D., Traverso L., Gnecchi Ruscone G.A., Peltola S., Salmela E., Neumann G.U. (2025). Ancient DNA connects large-scale migration with the spread of Slavs. Nature.

[83] Blöcher J., Vallini L., Velte M., Eckel R., Guyon L., Winkelbach L., Thomas M.G., Gharehbaghi N., Mitchell C.T., Schümann J. (2026). Demography and life histories across the Roman frontier in Germany 400–700 ce. Nature.

[84] Tian Y., Koncz I., Faragó N., Knipper C., Friedrich R., Vyas D.N., Samu L., Spekker O., Szeniczey T., Hajdu T. (2026). Unveiling the complexity of post-Roman polity formation in Pannonia using ancient DNA. Science.

[85] Vyas D.N., Koncz I., Modi A., Mende B.G., Tian Y., Francalacci P., Lari M., Vai S., Straub P., Gallina Z. (2023). Fine-scale sampling uncovers the complexity of migrations in 5th–6th century Pannonia. Curr. Biol..

[86] O’Leary N.A., Wright M.W., Brister J.R., Ciufo S., Haddad D., McVeigh R., Rajput B., Robbertse B., Smith-White B., Ako-Adjei D. (2016). Reference sequence (RefSeq) database at NCBI: current status, taxonomic expansion, and functional annotation. Nucleic Acids Res..

