# Supplementary material for: Paleogenomics reveals leprosy at the late antique burial site of Fallward in northern Germany
Source: iScience. 2026 Oct 15;29(11):117877. doi: 10.1016/j.isci.2026.117877 (PMC13691272; doi:10.1016/j.isci.2026.117877)
Supplement: Supplementary file 1 [file mmc1.pdf]

iScience, Volume *29*

Supplemental information

Paleogenomics reveals leprosy at the late antique burial site of Fallward in northern Germany

Daniel Anton Myburgh, Nicolas Antonio da Silva, Joanna H. Romeyer-Dherbey, Silke Grefen-Peters, Hauke Jöns, Christina Peek, Almut Nebel, and Ben Krause-Kyora

This PDF file includes:

Figure S1. Genetic sex determination of ancient samples

Figure S2. Outgroup f_3 statistics

Figure S3. f_4 statistics

Figure S4. Unsupervised admixture

Figure S5. Deamination plot of reads mapping to *Mycobacterium leprae* TN

Figure S6. Deamination plot of reads mapping against the human reference

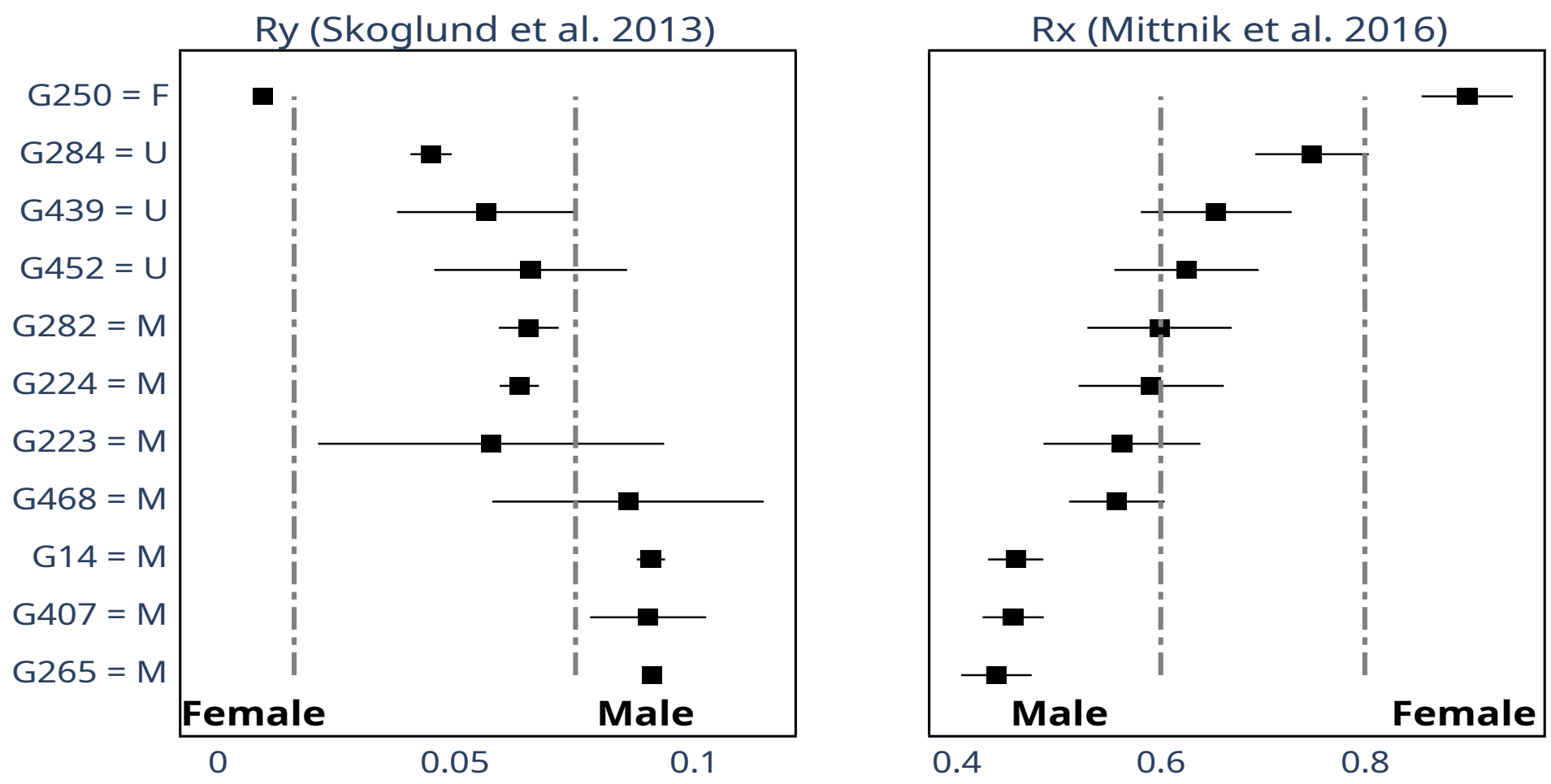

Figure S1. Genetic sex determination of ancient samples using two commonly applied methods: the Ry method **(A)** and the Rx method **(B)**. Panel A (Ry) shows the proportion of reads aligned to the Y chromosome relative to the total number of reads aligned to the autosomes, while Panel B (Rx) shows the proportion of reads aligned to the X chromosome relative to autosomal reads. These ratios are used to infer biological sex, with males expected to have higher Ry and lower Rx values, and females the opposite. Dashed lines along the y-axis in each panel indicate established threshold values for classifying individuals as genetically male or female. The error bars represent the 95% confidence intervals for each estimate, reflecting uncertainty due to read depth and sequencing variation.

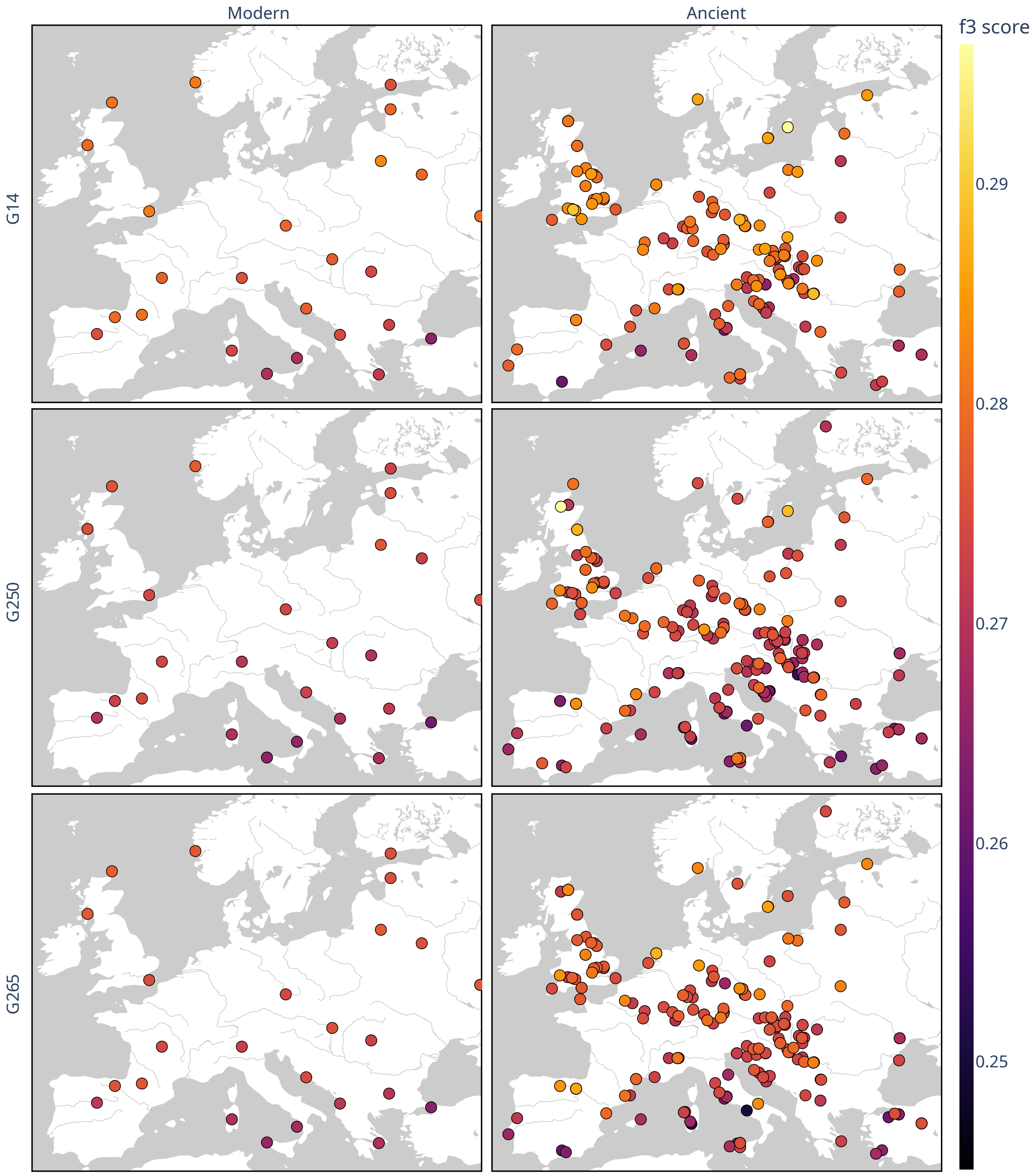

Figure S2. Outgroup f_3 statistics showing genetic affinities of the Fallward individuals included in population genetic analyses (rows) with published reference populations (columns). The statistic is calculated in the form f_3(Mbuti; X, Y), where X represents each Fallward sample and Y denotes the reference population. The left panels display affinities with modern west Eurasian populations from the Human Origins dataset, while the right panels show affinities with published ancient populations dating between 3000 and 1250 before present (BP). Higher

f_3 values indicate greater shared genetic drift between the Fallward individual and the tested population. Only tests with at least 10000 shared SNPs are shown.

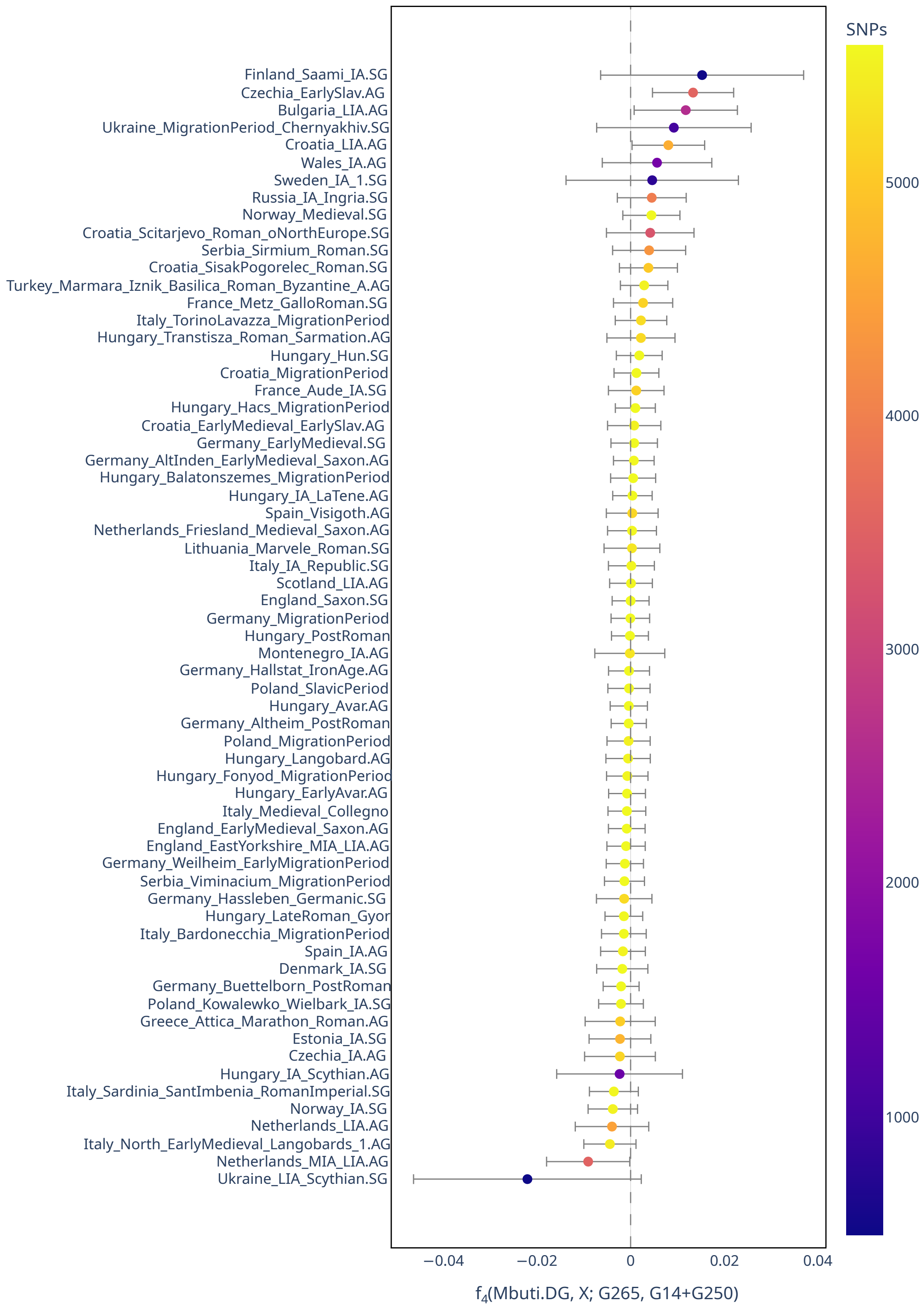

Figure S3. f_4 statistics of the form f_4(Mbuti.DG, X; G265, G14+G250), where X represents each ancient population included in the spatially and temporally relevant comparative dataset.

Error bars denote 95% confidence intervals. Colors indicate the number of overlapping single nucleotide polymorphisms used in each test.

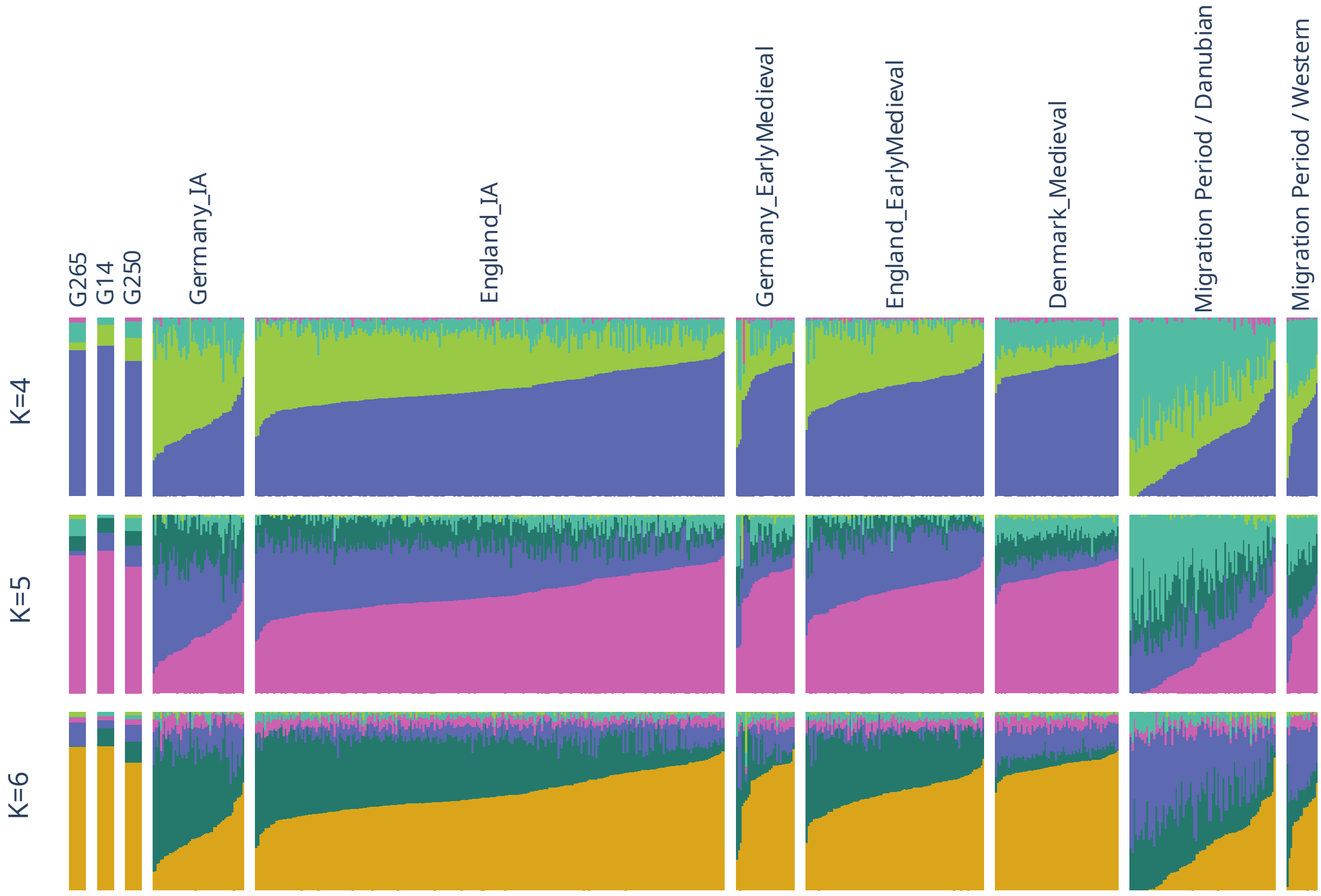

Figure S4. Unsupervised admixture results. Shown are the top three models (using K=4-6) with the lowest cross-validation error from all tested models (K=2-13) across 200 bootstraps.

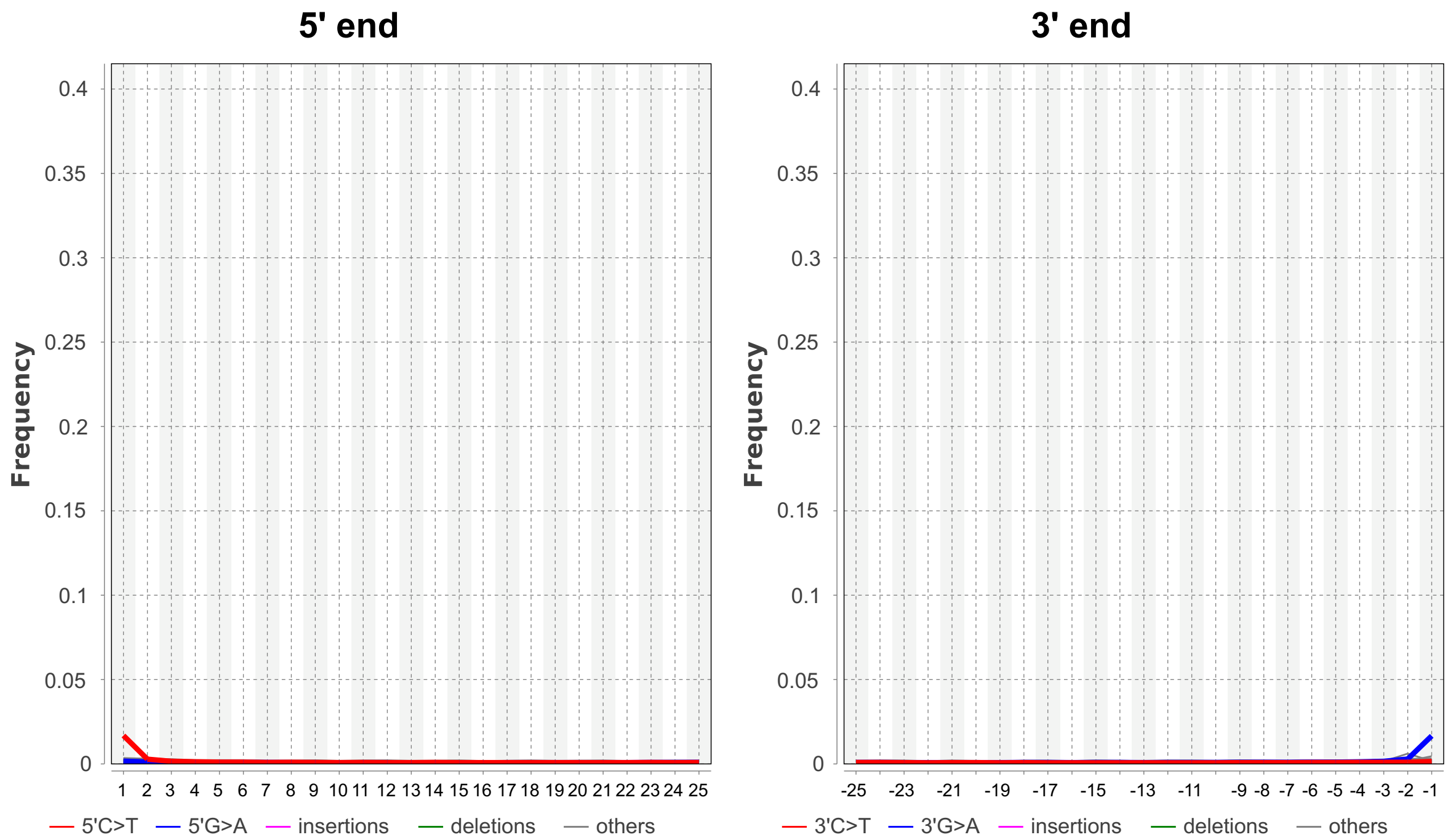

Figure S5. DNA deamination plot constructed using reads which were generated from the combined tooth and calcaneus bone taken from the individual buried in G265. Reads were mapped to *Mycobacterium leprae* TN.

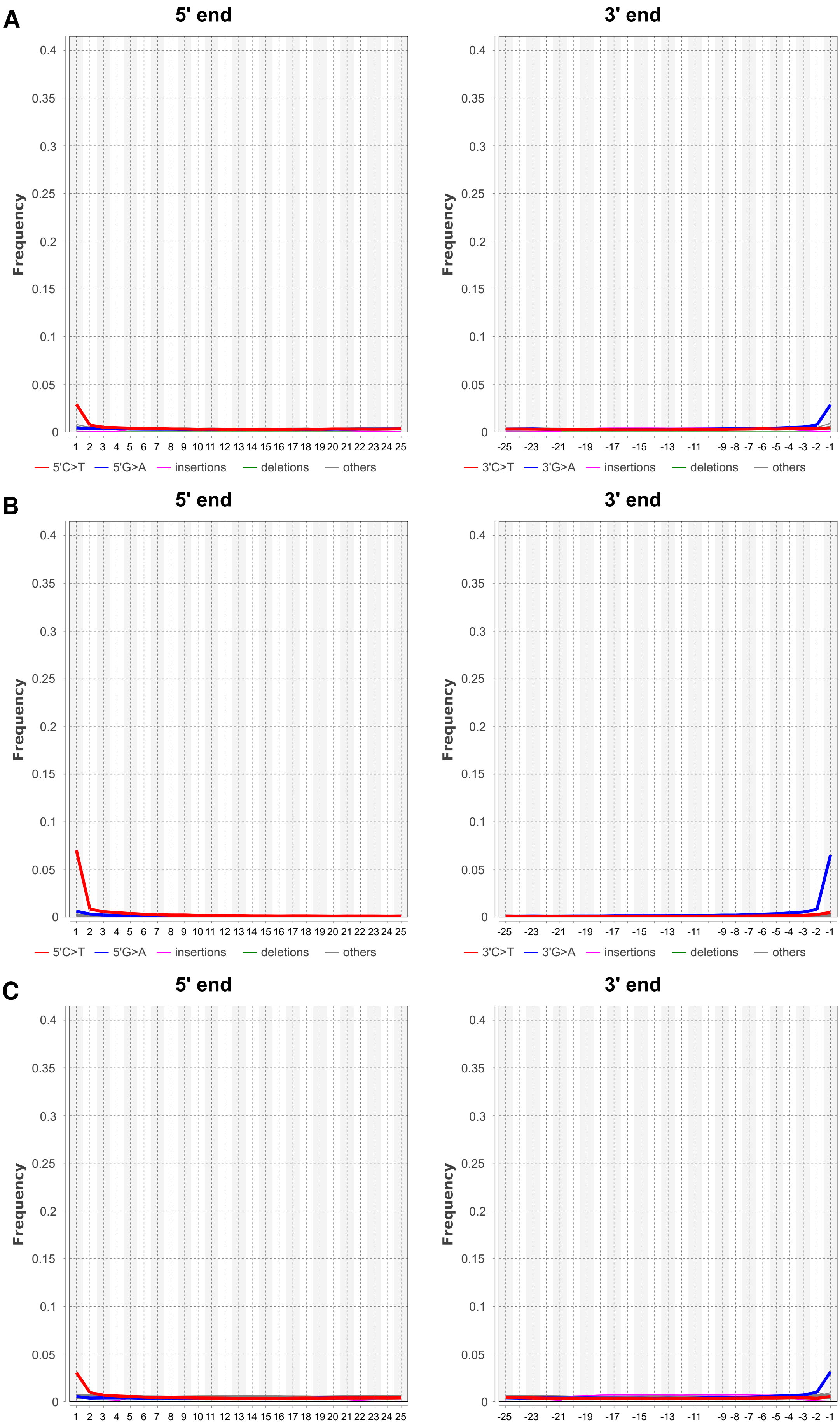
A
5' end
3' end
Frequency
0.4
0.35
0.3
0.25
0.2
0.15
0.1
0.05
0
1 2 3 4 5 6 7 8 9 10 11 12 13 14 15 16 17 18 19 20 21 22 23 24 25
-25 -23 -21 -19 -17 -15 -13 -11 -9 -8 -7 -6 -5 -4 -3 -2 -1
5'C>T
5'G>A
3'C>T
3'G>A
insertions
deletions
others
B
C

Figure S6. DNA deamination profiles for the three individuals included in population genetic analyses: G14 **(A)**, G250 **(B)**, and G265 **(C)**. Profiles are shown for reads mapped to the human reference genome (hg19), after duplicate removal and filtering for MAPQ>30.
